# Conventional and Novel Approaches to Establishing Mouse Models of Gastric Cancer in the Past, Present, and Potential Post-*H. Pylori* Infection Era

**DOI:** 10.1186/s12575-026-00333-5

**Published:** 2026-03-03

**Authors:** Hui Liu, Yunxiao Ge, Kangdong Liu, Zigang Dong

**Affiliations:** 1 Department of Pathophysiology, School of Basic Medical Sciences, College of Medicine, Zhengzhou University, Zhengzhou, Henan China; 2 China-US (Henan) Hormel Cancer Institute, No.127, Dongming Road, Jinshui District, Zhengzhou, Henan China; 3 Department of Physiology and Pharmacology, Karolinska Institutet, Stockholm, Sweden

**Keywords:** Gastric Cancer Mouse Model, Chemical Carcinogen, *H*. *Pylori*, Xenograft, Genetically Engineering

## Abstract

Gastric cancer remains a major cause of global cancer-related morbidity and mortality. Mouse models are indispensable tools for preclinical research into its mechanisms and therapies. Although *Helicobacter pylori* (*H. pylori*) infection is the primary risk factor for gastric cancer, developing mouse models based on this pathogen faces significant challenges. These include low bacterial colonization and survival rates, unpredictable and protracted tumorigenesis timelines, restrictions of host genetic backgrounds, the complexity of inflammatory and immune microenvironments, difficulties in standardized pathological assessment, and poor model reproducibility. In light of these limitations, research efforts have diversified into four principal categories of modeling approaches: chemical carcinogen-induced models, microbe-infected models (particularly those involving *H. pylori*), xenograft models, and genetically engineered mouse models. Each strategy offers distinct advantages and constraints, necessitating careful selection based on specific research objectives. This review comprehensively examines both conventional and emerging methods for establishing gastric cancer mouse models, situating them within a historical and evolving research landscape from past reliance on *H. pylori* to present and future approaches in the potential post-*H. pylori* infection era. We emphasize the applicability and compatibility of each modeling system with particular research goals, providing critical insights for selecting optimal in vivo platforms to advance the study of gastric carcinogenesis.

## Introduction

Gastric cancer remains one of the most malignant cancer types worldwide, ranking the sixth in incidence rate and seventh in mortality globally in 2022 according to International Agency for Research on Cancer (IARC) of World Health Organization (WHO). The risk factors of gastric cancer mainly include *Helicobacter pylori* (*H. pylori*) infection, Epstein-Barr virus (EBV) infection, age and gender, family history, alcohol consumption, smoking, obesity, as well as stress [1], yet preclinical research into its control remains constrained by limitations in in vivo models with gastric cancer mouse models being the most widely used tool, encompassing chemical carcinogen-induced, microbe-infected (predominantly *H. pylori*), xenograft, and genetically engineered models.

**Fig. 1:**
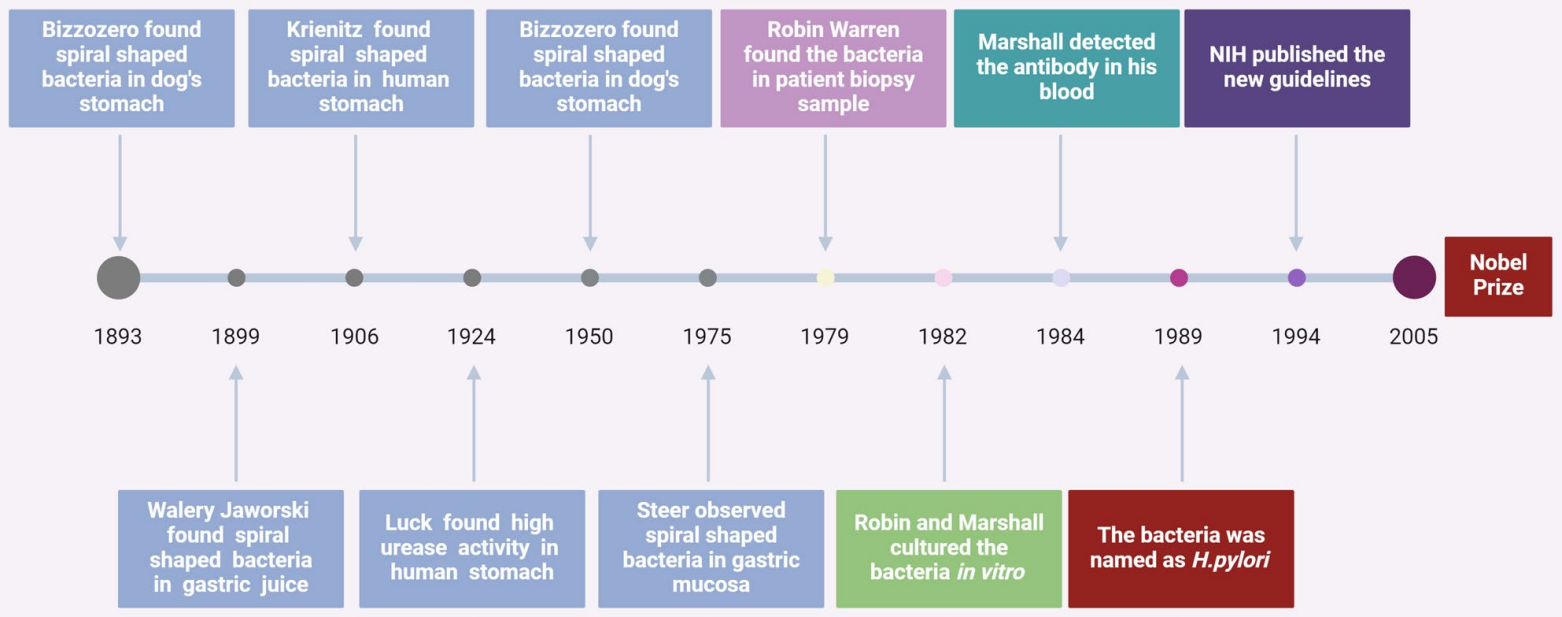
The discovery and research process of *Helicobacter pylori*. The century-long elucidation of *Helicobacter pylori* commenced with Giulio Bizzozero’s landmark 1893 observation of spiral bacteria in canine gastric mucosa. This discovery initiated a cascade of scientific inquiry: Krénítz replicated these findings in dogs (1899), Bizzozero subsequently identified analogous organisms in human stomachs (1906), and Walery Jaworski’s 1924 documentation of spiral bacteria in gastric secretions further solidified microbial presence. Critical breakthroughs emerged in 1982 when Robin Warren and Barry Marshall achieved in vitro cultivation, conclusively demonstrating bacterial viability. Subsequent milestones included definitive taxonomic classification as *Helicobacter pylori* (1989), NIH consensus guidelines establishing clinical management protocols (1994), and the ultimate scientific validation through the 2005 Nobel Prize in Physiology or Medicine. This process laid an important foundation for the understanding and treatment of gastric diseases in the future

*H. pylori* is the Class I carcinogen for gastric cancer verified with a long history (Fig. 1). *H. pylori* infection models are predominantly established in rodents owing to their high fecundity, well-defined genetic backgrounds, and stable quality control, whereas other animals like primates and ferrets are limited by high natural infection rates, high costs, and uncontrollable variables [2–5]. Among rodents, rats exhibit poor *H. pylori* colonization and mild inflammation, guinea pigs develop gastritis restricted to non-fundic regions, and Mongolian gerbils require long modeling timelines (> 62 weeks) with low gastric cancer incidence (~ 37%), driving the growing adoption of *H. pylori*-infected mouse models for their shorter cycles and higher tumor rates [6, 7] particularly when combined with co-factors (chemical carcinogens, alcohol, high salt, capsaicin, stress) to mimic human multi-factorial carcinogenesis [8–12]. Additionally, emerging models like *Streptococcus anginosus*-induced gastric cancer have expanded the microbial modeling landscape, while chemical carcinogen, xenograft, and genetically engineered models have become routine tools [13, 14].

This review systematically summarizes methods for establishing gastric cancer mouse models across historical, current, and potential post-*H. pylori* infection eras, with a focus on microbe-induced models, especially *H. pylori* and its combinations with co-factors, and a selective overview of key techniques, aiming to assist researchers in selecting optimal in vivo platforms for gastric cancer studies by clarifying the utility of each model within the evolving research landscape.

## Chemical Carcinogen Induced Gastric Cancer Mouse Model

**Fig. 2:**
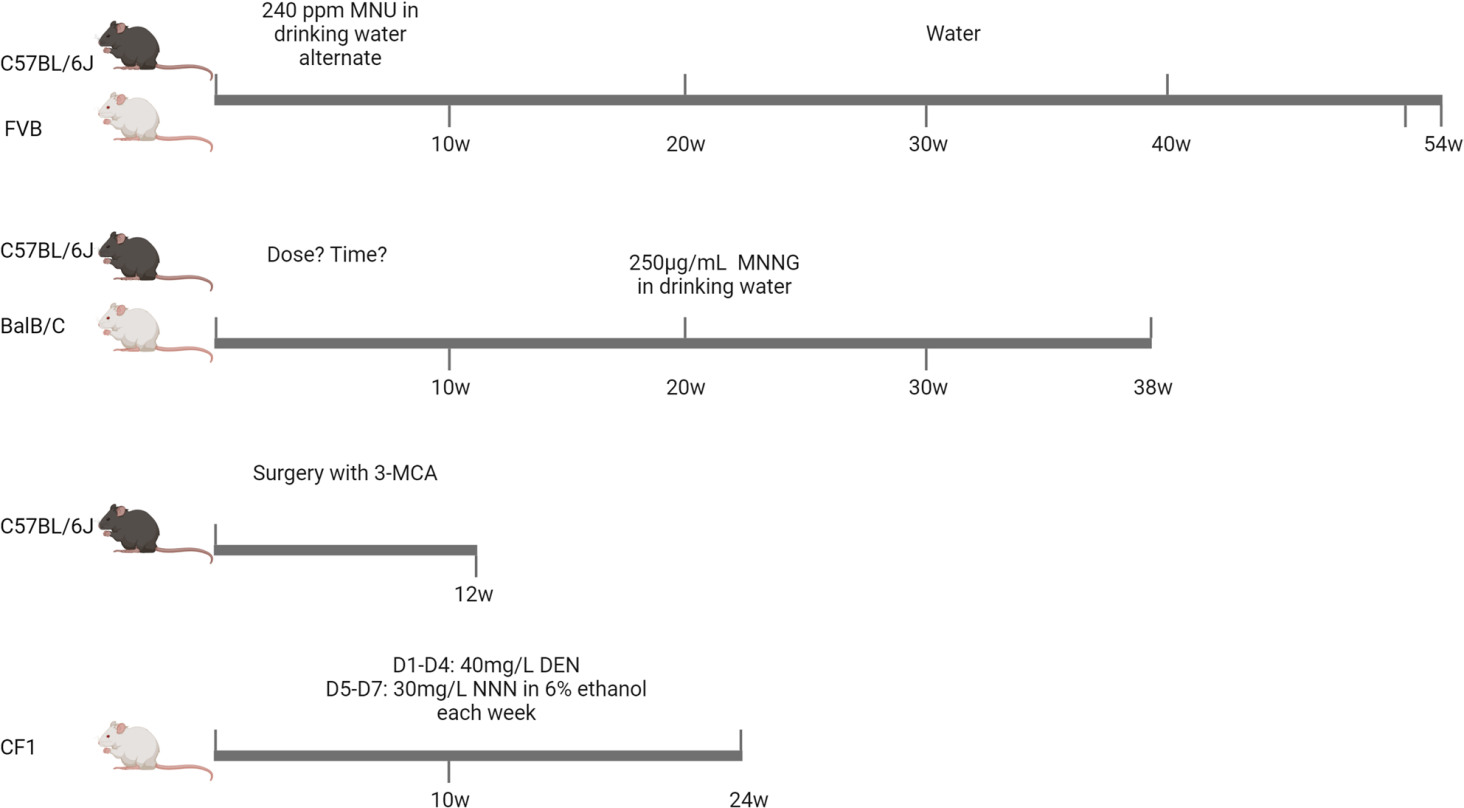
Chemical carcinogen induced gastric cancer mouse models. Different strains of mice (C57BL/6J, FVB, BALB/c, CF1) and different carcinogens are used to construct different gastric cancer models. C57BL/6J and FVB strains were alternately given 240 ppm MNU in drinking water for a period of time before switching to drinking water; C57BL/6J and BALB/c mice were given 250 µg/mL MNNG in drinking water; C57BL/6J mice underwent surgery and were treated with 3-MCA; CF1 strain mice were given 40 mg/L DEN on days 1–4 and 30 mg/L NNN in 6% ethanol on days 5–7, weekly, followed by drinking water

Chemical carcinogen-induced gastric cancer mouse models serve as pivotal tools for dissecting environmental drivers of gastric carcinogenesis, such as dietary nitrosamines or pollutant exposure, by recapitulating the multi-stage progression of human disease from chronic gastritis to invasive carcinoma under controlled experimental conditions. The core objective of this section is to systematically outline the design, performance, and translational utility of these models, while clarifying their strengths and inherent limitations to guide rational model selection (Fig. 2).

Key carcinogens employed include N-methyl-N-nitrosourea (MNU), N-methyl-N’-nitro-N-nitrosoguanidine (MNNG), 3-methylcholanthrene (3-MCA), and a synergistic combination of diethylnitrosamine (DEN), N’-nitrosonornicotine (NNN), and ethanol [15–18]. Each exhibits distinct modes of action that dictate its suitability for addressing specific research questions, such as large-scale drug screening or early-stage carcinogenesis studies. MNU is the most widely used carcinogen for gastric cancer modeling. For 6-week-old C57BL/6J or FVB mice, MNU is consistently administered at 240 ppm in light-shielded drinking water on alternate weeks for 10 weeks, followed by regular water until week 54 [19–21]. This regimen induces gastric cancer in both strains with minimal inter-experimental variability: tumor incidence averages 60–70% at 54th week, with pathological features (glandular dysplasia, invasive adenocarcinoma) that align with human intestinal-type gastric cancer. A key strength of MNU models lies in their broad adaptability to common inbred strains, enabling large-scale studies such as high-throughput drug screening or oncogene validation. However, these models have notable limitations that they lack the microbial-immune crosstalk inherent to human gastric cancer, and their long timeline (≥ 54 weeks) increases experimental cost and attrition rates.

In contrast to MNU’s stability, MNNG-based models are characterized by unresolved debates regarding optimal dosing and timeline, reflecting strain-specific differences in sensitivity. For example, 150 µg/mL MNNG in free drinking water induces gastric cancer in C57BL/6J mice at 8th week, C57BL/6JGpt mice at 18th week, and BALB/C mice at 38th week [22–25]. A separate study reported tumor induction in C57BL/6J mice using 100 mg/mL MNNG (administered on alternate weeks for 20 weeks), a 667-fold difference in concentration that highlights inconsistencies limiting cross-study comparison [26]. Notably, recent work has refined MNNG’s utility beyond end-stage cancer. 250 µg/mL MNNG in drinking water reliably induces chronic atrophic gastritis (CAG), a well-established precancerous lesion, in BALB/C mice after 12 weeks [27]. This finding does not position MNNG as a superior alternative to MNU because the primary limitation of MNNG models remains the lack of standardized protocols, which complicates reproducibility across laboratories.

3-MCA-induced models uniquely enable site-specific carcinogenesis, addressing a gap in simulating the anatomical predilection of human antral gastric cancer. The protocol requires surgical implantation. A sterile cotton thread knot impregnated with 1 mg of 3-MCA dissolved in DMSO is inserted into the lesser curvature of the gastric antrum from mucosal to serosal layer via an incision in the greater curvature; a second knot is tied to the serosal surface to secure the thread, followed by a figure-of-eight suture to embed the serosal knot, and closure of the stomach wall and abdominal incisions. 12 weeks post-surgery, C57BL/6J mice develop into gastric cancer [28]. The surgical nature of 3-MCA models, however, limits their scalability compared to MNU or MNNG. The procedure requires specialized technical expertise, and post-surgical complications can increase mouse mortality, reducing overall experimental efficiency.

The combination of DEN, NNN, and ethanol leverages synergistic carcinogenic effects to optimize tumor incidence. Particularly, over 24 weeks, CF1 mice receive 40 mg/L DEN in drinking water for the first 4 consecutive days of each week, with the remaining 3 days dedicated to a solution containing 30 mg/L NNN and 6% ethanol [15]. DEN initiates DNA damage via alkylation, NNN targets gastric epithelial cells to amplify mutagenesis, and ethanol exacerbates tissue injury by disrupting mucosal barrier function, collectively yielding a gastric cancer incidence of ~ 85% at 24th week, higher than any single agent alone. A key limitation of this combination model is its narrow strain applicability. It has only been validated in CF1 mice, restricting its use in studies requiring genetic manipulation such as crossbreeding with transgenic strains common in C57BL/6J backgrounds. All chemical carcinogen protocols conclude with standardized downstream processing. Mice are euthanized via cervical dislocation, stomachs are opened along the greater curvature for macroscopic tumor assessment, and tissues are fixed in 10% buffered formalin for histological analysis or snap-frozen for molecular profiling.

Collectively, chemical carcinogen models excel at recapitulating environmental etiologies of human gastric cancer, but their utility depends on aligning model selection with research goals to avoid overgeneralization. MNU models are preferred for large-scale, reproducible studies like drug screening due to their strain adaptability and stable tumorigenesis; MNNG models are valuable for investigating early precancerous progression, provided that strain-specific dosing is clearly reported; 3-MCA models enable site-specific mechanism research, albeit with higher technical demands; and the DEN-NNN-ethanol combination optimizes efficiency for studies requiring rapid tumor development. A universal limitation of these models is their inability to fully mimic the multi-factorial etiology of human gastric cancer, as they lack the microbial like *H. pylori* and immune components that drive most clinical cases, and thus should be complemented with microbe-co-culture or immunocompetent models when studying inflammation-carcinogenesis crosstalk.

## Microbes Infection Induced Gastric Cancer Mouse Model

The core objective of this section is to systematically assess mouse models that recapitulate microbial-driven gastric carcinogenesis with a focus on *H. pylori* and emerging non-*Helicobacter* pathogens, while clarifying their ability to mimic human disease, technical constraints, and translational relevance. These models fill a critical gap left by chemical or genetic models alone. They capture the inflammation-carcinogenesis cascade inherent to human gastric cancer, where chronic microbial colonization disrupts mucosal homeostasis, fuels persistent inflammation, and drives stepwise pathological progression from gastritis to adenocarcinoma. Below, we detail the design, performance, and limitations of each microbial model, with explicit alignment to research goals to minimize redundancy and guide rational model selection.

### *Helicobacter Pylori* Infection Induced Gastric Cancer Mouse Model

*H. pylori*-infected mouse models effectively recapitulate the Correa cascade of human gastric carcinogenesis, where bacterial virulence, host genetics, and environmental cues converge to drive carcinogenesis, while enabling mechanistic exploration of microbe-host interactions [29]. *H. pylori* is a well-validated Class I carcinogen with over half of the global population infected and infection rates vary by region, exceeding 80% in developing areas and remaining below 40% in developed countries [30, 31]. As the leading risk factor for gastric cancer, *H. pylori*-driven models fill a critical gap in simulating the chronic inflammation-malignant transformation cascade that underpins most clinical cases, making them indispensable for studying infection-associated carcinogenesis.

The tumorigenic potential of *H. pylori* in mice is shaped by bacterial virulence, host genetics, and environmental cues, though quantifying the exact timeline of tumorigenesis for specific strains remains challenging due to the complexity of these interactions. Bacterial virulence is primarily dictated by genes such as CagA (cytotoxin-associated gene A) and VacA (vacuolating cytotoxin gene A). Strains harboring both genes like CagA^+^/VacA s1/m1 are more likely to induce severe gastric pathology, including adenocarcinoma [32]. Host factors further modulate susceptibility, for instance, C57BL/6J mice exhibit distinct immune responses to infection and are widely used due to the availability of genetic variants, while Mongolian gerbils are preferred for their high sensitivity to *H. pylori* but suffer from prolonged modeling timelines (> 62 weeks) and low gastric cancer incidence (~ 37%) when infected alone. Environmental variables such as diet (high salt, alcohol) or chemical co-carcinogens (MNU) can synergistically enhance tumorigenesis, though these modifiers also introduce variability that complicates the quantification of strain-specific tumorigenic rates.

Beyond *H. pylori*, non-*H. pylori* helicobacters (NHPH) have emerged as significant gastric pathogens in humans, demonstrating comparable pathogenic potential in inducing gastroduodenal disorders [33, 34]. Notably, *Helicobacter suis* (*H. suis*) represents the predominant NHPH species colonizing the human stomach [35]. Experimental infection of C57BL/6J mice, BALB/c mice, or Mongolian gerbils with *H. suis* induces pathological features that mirror human disease, including chronic gastritis, parietal cell degeneration, and mucosa-associated lymphoid tissue (MALT) lymphoma-like lesions (observed in Mongolian gerbils at 8 months) [35, 36]. Other NHPH strains, such as *H. Heilmanii* and *H. felis*, exhibit varying pathogenicity [37]. For example, *H. felis* strongly induces gastritis and gastric cancer in mice serving as a substitute for *H. pylori* in some studies, while *H. salomonis* fails to colonize mice entirely, highlighting strict host tropism [38–40]. These NHPH models expand the microbial modeling landscape but require careful strain selection, as their lack of canonical virulence genes necessitates investigation of alternative pathogenic mechanisms. We have summarized the biological characteristics, host range, disease associations, and experimental models of various *Helicobacter* strains, providing reference for studying their pathogenic mechanisms and host interactions (Table 1).

**Table 1:** Comparison of *helicobacter* strain characteristics and applications

Strain Name		Characteristics	Source	Host	Disease Association	Experimental Model	Reference
*H. pylori*	*NSH79*	Mouse-adapted strain, partial loss of cag PAI function, low IL-8 induction ability	Human gastric mucosa	Mouse	Gastritis, peptic ulcer, gastric cancer	C57BL/6 mouse model	[41]
	*NSH57*	Mouse-adapted strain, intact cag PAI function, high IL-8 induction ability	Human gastric mucosa	Mouse	Gastritis, peptic ulcer, gastric cancer	C57BL/6 mouse model	[41]
	*X47*	Mouse-adapted strain, lacks cag PAI	Human gastric mucosa	Mouse	Gastritis, peptic ulcer, gastric cancer	C57BL/6 mouse model	[42]
	*X47-2AL*	CagA-negative, VacA-positive	Clinical sample	Mouse	Gastritis, gastric ulcer, gastric cancer	Adult mouse model	[43, 44]
	*B128*	Mouse-adapted strain, CagA^+^, VacA s1a, associated with gastric ulcer	Human gastric mucosa	Mouse	Gastritis, peptic ulcer, gastric cancer	C57BL/6 mouse model	[42, 45]
	*SS1*	Mouse-adapted strain, CagA^+^, VacA^+^, partial loss of cag PAI function	Human gastric mucosa	Mouse	Gastritis, peptic ulcer, gastric cancer	C57BL/6 and BALB/c mouse models; Mongolian gerbils	[35, 43, 46–51]
	*245*	Intact cag PAI function, high IL-8 induction ability	Human gastric mucosa	Mouse	Gastritis, peptic ulcer, gastric cancer	C57BL/6 mouse model	[47]
	*251*	Intact cag PAI function, high IL-8 induction ability	Human gastric mucosa	Mouse	Gastritis, peptic ulcer, gastric cancer	C57BL/6 mouse model	[47]
	*PMSS1*	Mouse-adapted strain, CagA^+^, VacA^+^, pathogenic partial loss of cag PAI function	Human gastric mucosa	Mouse	Gastritis, peptic ulcer, gastric cancer	C57BL/6 and BALB/c mouse models	[35, 52, 53]
	*AM1*	Lacks cag PAI contains kanamycin resistance marker	Clinical sample	Mouse	Gastritis, gastric ulcer, gastric cancer	Adult mouse model	[43, 48]
	*AL10103*	Contains kanamycin resistance	Clinical sample	Mouse	Gastritis, gastric ulcer, gastric cancer	Adult mouse model	[43]
	*SS2000*	Lacks cag PAI	Clinical sample	Mouse	Gastritis	Adult mouse model	[54]
	*10,319*	Clinical isolate	Clinical sample	Mouse	Gastritis, gastric ulcer, gastric cancer	Adult mouse model	[55]
	*10,217*	Clinical isolate	Clinical sample	Mouse	Gastritis, gastric ulcer, gastric cancer	Adult mouse model	[55]
	*26,695*	Sequenced clinical isolate	Clinical sample	Mouse	Gastritis, gastric ulcer, gastric cancer	Adult mouse model	[55]
	*J99*	Sequenced clinical isolate, CagA^+^, VacA^+^, associated with corpus-predominant gastritis and hypochlorhydria	Clinical sample	Mouse; Mongolian gerbil	Gastritis, gastric ulcer, gastric cancer	Mouse, Mongolian gerbil model	[55–57]
	*94–2728*	Isolated from a cat with gastritis	Cat	Cat	Feline gastritis	Cat model	[58]
	*UA948*	Expresses Lea and Lex	Clinical sample	Mouse	Gastritis, gastric ulcer, gastric cancer	Adult mouse model	[59]
	*UA861*	Expresses α-glucosyl polyLacNAc	Clinical sample	Mouse	Gastritis, gastric ulcer, gastric cancer	Adult mouse model	[59]
	*UA1258*	Expresses Ley	Clinical sample	Mouse	Gastritis, gastric ulcer, gastric cancer	Adult mouse model	[59]
	*UA802*	Expresses Ley	Clinical sample	Mouse	Gastritis, gastric ulcer, gastric cancer	Adult mouse model	[59]
	*UA1264*	Does not express complete Lewis’s antigen	Clinical sample	Mouse	Gastritis, gastric ulcer, gastric cancer	Adult mouse model	[59]
	*HP238*	CagA-positive, associated with IFN-γ signaling inhibition	Clinical isolate	Human gastric epithelial cells and mouse gastric epithelial cells	Gastritis, Gastric cancer	Human gastric epithelial cell lines and mouse gastric epithelial cells	[60]
	*HP238CagAm*	CagA-deficient, used to study the role of CagA in IFN-γ signaling inhibition	HP238 CagA gene mutant strain	Human gastric epithelial cells and mouse gastric epithelial cells	Gastritis, Gastric cancer	Human gastric epithelial cell lines and mouse gastric epithelial cells	[60]
	*HPAG1*	Parental strain, less pathogenic compared to SS1	Laboratory culture	C57BL/6J female chimeric mice	Chronic gastritis	C57BL/6J female chimeric mice	[61]
	*HPARE*	Adapted to mouse models, highly pathogenic	Laboratory culture	C57BL/6J female chimeric mice	Chronic gastritis, Gastric cancer	C57BL/6J female chimeric mice	[61]
	*clinical isolates*	Carry babA2, VacA, and CagA genes, associated with gastric ulcers and gastric cancer	German population	Humans	Gastric ulcers, Gastric cancer	Human clinical samples	[62]
	*G1.1*	CagA^+^, VacA s1a, associated with duodenal ulcer	Clinical isolate	Mouse	Duodenal ulcer	Mouse model	[45]
	*G27*	CagA^+^, VacA^+^, pathogenic	Clinical isolate	Mouse	Gastritis	Mouse model	[52]
	*G27 ΔCagE*	CagA^−^, T4SS non-functional	G27 mutant	Mouse	None	Mouse model	[52]
	*G27 ΔCagA*	CagA^−^, T4SS functional	G27 mutant	Mouse	None	Mouse model	[52]
	*119p*	CagA-positive, VacA-positive	Human duodenal ulcer patient	Mouse	Peptic ulcer disease	Mouse model for long-term infection	[63]
	*G50*	CagA-negative, VacA-negative	Not explicitly stated	Mouse	Not associated with severe disease	Mouse model for low virulence	[63]
	*ATCC 43,504*	Not explicitly stated	Not explicitly stated	Mouse	Lupus exacerbation	FcγRIIb-deficient lupus mouse model	[64]
	*TN2GF4*	CagA+, VacA+	Gastric ulcer patient	Mongolian gerbil	Gastric cancer	Mongolian gerbil model	[46]
	*7.13*	CagA+, VacA+	Duodenal ulcer patient	Mongolian gerbil	Lower gastric cancer risk	Mongolian gerbil model	[46]
	*SC4*	CagA+, VacA+	Clinical isolate	Human	Gastric cancer	Human samples	[57]
	*D1*	CagA+, VacA+	Clinical isolate	Human	Gastric cancer	KRAS1 mouse model	[65]
	*C2*	CagA+, VacA+	Clinical isolate	Human	Gastric cancer	KRAS1 mouse model	[65]
*H. suis*	*HsMmR08041a*	Associated with monkey gastritis	Monkey gastric mucosa (Crab-eating macaque)	BALB/c mice and Mongolian gerbils	Gastritis	BALB/c mice and Mongolian gerbils	[66]
	*HsMf505/1*	Unable to colonize Mongolian gerbils	Monkey gastric mucosa (Rhesus macaque)	BALB/c mice and Mongolian gerbils	Gastritis	BALB/c mice and Mongolian gerbils	[66]
*H. felis*	*ATCC 49,179*	Associated with chronic gastritis and gastric cancer, no CagA and VacA genes	Laboratory culture	C57BL/6 mice and Mongolian gerbils	Chronic gastritis, Gastric cancer	C57BL/6 mice and Mongolian gerbils	[49, 67, 68]
	*CCUG 37,471*	No CagA and VacA genes	Cat	Mouse	Gastritis	Mouse inflammation model	[68]
	*CS1*	No CagA and VacA	Cat gastric mucosa	Mouse	Low gastric cancer risk	Mouse model	[69]
*H. bizzozeronii*	*CCUG 35,545*	No CagA and VacA genes	Dog	Mouse	Gastritis	Mouse inflammation model	[68]
*H. salomonis*	*CCUG 37,845*	No CagA and VacA genes	Dog	Mouse	Not associated with detectable infection	Mouse inflammation model	[68]

To construct *H. pylori*-induced gastric cancer mouse model, several key steps must be followed. First, select the mouse strain. Murine susceptibility to *H. pylori* infection exhibits significant inter-strain variation, with differential inflammatory and immunological outcomes observed across genetically distinct mouse models [70]. C57BL/6J mice are ideal for studies requiring genetic manipulability and moderate tumor incidence, while Mongolian gerbils are suited for their high sensitivity to *H. pylori* infection and increased risk of developing cancer [71, 72]. Second, *H. pylori* strain choice is equally pivotal. Strains with higher colonization ability, such as those expressing specific virulence factors, are preferred [73]. Mouse-adapted strains such as *PMSS1* exhibit stable long-term colonization (85–95% in C57BL/6J mice), with 40–50% of mice developing adenocarcinoma after 18 months of single infection and more than 70% developing tumors within 12 months when combined with MNU [74]. The *SS1* strain requires frequent passaging to maintain colonization (70–80% infection rate) and induces dysplasia in less than 10% of wild-type mice after 24 months, though its tumorigenicity rises to 90% in INS-GAS transgenic mice [46]. The *7.13* strain, while highly colonizing (nearly 100% short-term), causes premature mouse death due to high toxicity, limiting its utility. Third, inoculation and validation protocols must be standardized. Oral gavage of *H. pylori* suspensions (typically 1 × 10⁸ − 1 × 10⁹ CFU/mL, repeated 3–5 times) is common, followed by confirmation of chronic infection via rapid urease tests or Giemsa staining, and longitudinal sampling at 10th, 25th, 45th, or 72nd week to monitor progression from chronic gastritis to adenocarcinoma [29, 71, 72].

The *H. pylori* driven gastric cancer mouse model is valuable because it reproduces the natural continuum of human gastric carcinogenesis from chronic inflammation to malignant transformation under a biologically relevant infectious trigger. However, it is constrained by limitation that tumorigenic timelines are unpredictable and protracted (12–24 months for mice, more than 62 weeks for gerbils), making high-throughput studies impractical. Additionally, the model cannot fully recapitulate human interindividual variability in immune responses or co-morbidities, further restricting its translational scope.

### Combination of *H. pylori* with other Factors Induced Gastric Cancer Mouse Model

Human gastric cancer is rarely driven by *H. pylori* alone, instead, it arises from synergies between infection and environmental factors or host stress. The goal of combined models is to replicate this multi-factorial etiology, accelerate carcinogenesis, and uncover interactive mechanisms between *H. pylori* and co-insults, addressing the limitation of single-infection models which often require more than 18 months to induce cancer.

#### *H. pylori* & Chemical Carcinogen

The *H. pylori*-chemical carcinogen co-induced gastric cancer mouse model bridges the gap between single-factor experimental settings and clinical reality, making it an essential tool for exploring the interactive effects of microbial and environmental factors in accelerating carcinogenesis.

MNU is a commonly used chemical carcinogen in gastric cancer mouse model. Studies have revealed the combination of MNU treatment and *H. pylori* infection significantly increased the incidence of gastric cancer compared to either treatment alone [46, 75, 76]. Experimental protocols for this model vary by animal strain but follow consistent logic of carcinogen priming combined with *H. pylori* infection, with precise reporting critical to reproducibility.

For C57BL/6J mice, a typical protocol involves 150–240 ppm MNU in drinking water biweekly for 10–12 weeks, followed 1 week later by oral gavage of *H. pylori* (1 × 10⁹ CFU/mL, 3–9 doses on alternate days). Euthanasia occurs at 50-52nd week, with tumor incidence ranging from 40 to 75% depending on MNU dose and bacterial strain [76–81]. Apart from *H. pylori*, the combination of *H. felis* and MNU is a powerful model for studying gastric carcinogenesis in mice [82].

BALB/c mice have also been used to study the occurrence and development of gastric cancer. Six- to eight-week-old SPF-grade BALB/c male mice were administered 0.4 mL of *H. pylori* solution following a 0.2 mL dose of 2% NaHCO_3_ solution, repeated every 2 days for a total of five times. After *H. pylori* infection, mice were allowed to freely drink a 240 ppm MNU solution and received 0.03% ranitidine in their diet every 2 days, followed by a 1-day fast, for 20 weeks [83].

**Fig. 3:**
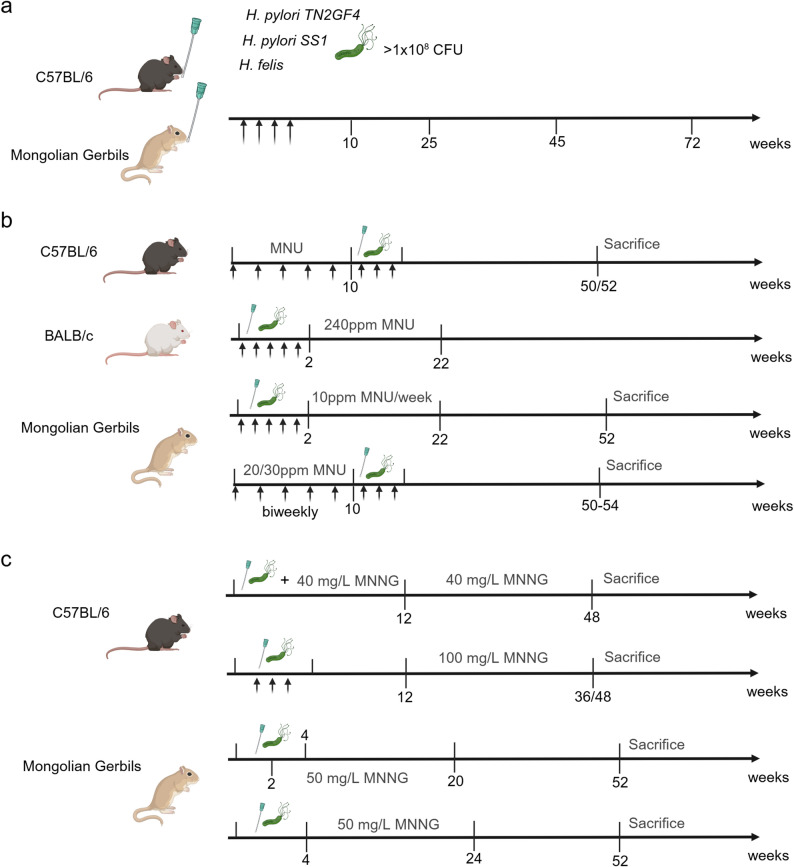
*H. pylori* or *H. pylori* combined with chemical carcinogen induced gastric cancer mouse models. (**a**) *H. pylori*; (**b**) *H. pylori* combined with MNU; (**c**) *H. pylori* combined with MNNG

Mongolian gerbils are also a used host for studying *H. pylori*-MNU co-induced gastric carcinogenesis, with experimental protocols differing primarily in MNU concentration, treatment duration, and the sequence of *H. pylori* infection relative to MNU exposure, variables that directly impact tumor incidence but have not yet been standardized. For instance, 6-week-old male gerbils fasted for 24 h receive oral gavage of *H. pylori* (1 × 10⁸ CFU/mL, 1 mL), resume feeding after 4 h, maintain infection for 2 weeks, then undergo 20 weeks of 10 ppm MNU treatment, with euthanasia at week 52 [84, 85]. In contrast, 7-week-old gerbils receive 20 ppm MNU in drinking water every 2 weeks for five cycles; 1 week after MNU completion, they are infected with *H. pylori ATCC43504* (CagA^+^/VacA^+^, 1 × 10^8^ CFU/mL) via three alternate-day gavages and euthanized at week 50 [86, 87]. A third protocol uses 5-week-old male gerbils. 30 ppm MNU is administered in drinking water on alternate weeks for five exposures, followed by oral gavage of *H. pylori* (1 × 10⁹ CFU/mL), with euthanasia at week 54 [88]. Collectively, these data confirm that MNU (10–30 ppm) combined with *H. pylori* effectively induces gastric cancer in gerbils, but critical gaps remain, specifically, which MNU concentration and duration yield the highest, most reproducible tumor incidence, and whether the sequence of *H. pylori* infection and MNU treatment modulates carcinogenic synergy. Sugiyama et al. [89] investigated the impact of *H. pylori* infection on gastric cancer development using 170 male Mongolian gerbils, aged 7 weeks, divided into nine groups of 18–20 animals each. The study comprised two experiments. In Experiment I, four groups received *H. pylori* infection followed by administration of MNU in drinking water at 10 ppm or 3 ppm for 20 weeks. In Experiment II, four groups underwent MNU treatment (30 ppm for 6 weeks or 10 ppm for 10 weeks) and were infected with *H. pylori* one week later. All gerbils were euthanized at week 40. The findings indicated that *H. pylori* infection, whether before or after MNU exposure, significantly increased the incidence of gastric adenocarcinoma, highlighting its role as a promoter of gastric cancer (Fig. 3b).

Beyond MNU, *H. pylori* has also been shown to enhance gastric mucosal cell sensitivity to MNNG, another nitrosamine carcinogen, with protocols tailored to both C57BL/6J mice and Mongolian gerbils [90]. In C57BL/6J mice, 8-week-old females receive *H. pylori SS1* (10⁷ CFU/mouse) via alternate-day oral gavage (five treatments/month) concurrent with 40 mg/L MNNG in drinking water; after 3 months of co-treatment, mice continue on MNNG alone until euthanasia at 48th week [91]. A separate study uses 6-week-old C57BL/6J mice. Three alternate-day gavages of a mixed *H. pylori* strain suspension (1 × 10⁹ CFU/mL) are administered, and 3 months after the final gavage, infected mice receive 100 mg/L MNNG (refreshed every 2 days) until euthanasia at 32 or 44 weeks post-infection [92]. In Mongolian gerbils (5–8 weeks old), oral inoculation of *H. pylori* (9 × 10⁸ CFU/mL, 2 mL) twice weekly for 4 weeks is paired with 50 mg/L MNNG in drinking water from week 2 to 20, followed by distilled water until euthanasia at week 52 [93]. Similarly, 5-week-old male gerbils are gavaged with *H. pylori ATCC 43,504* (10⁹ CFU/mL, 1 mL), allowed food/water access after 12 h, then receive 50 mg/L MNNG three times weekly for 20 weeks starting after 4 weeks post-infection; euthanasia occurs after 16, 24, or 52 weeks to assess histological progression and cell proliferation [94, 95]. These protocols confirm that *H. pylori*-MNNG co-induction recapitulates multifactorial carcinogenesis across species, though strain-specific differences in MNNG sensitivity require careful consideration for cross-study reproducibility (Fig. 3c).

#### *H. pylori* & Alcohol

**Fig. 4:**
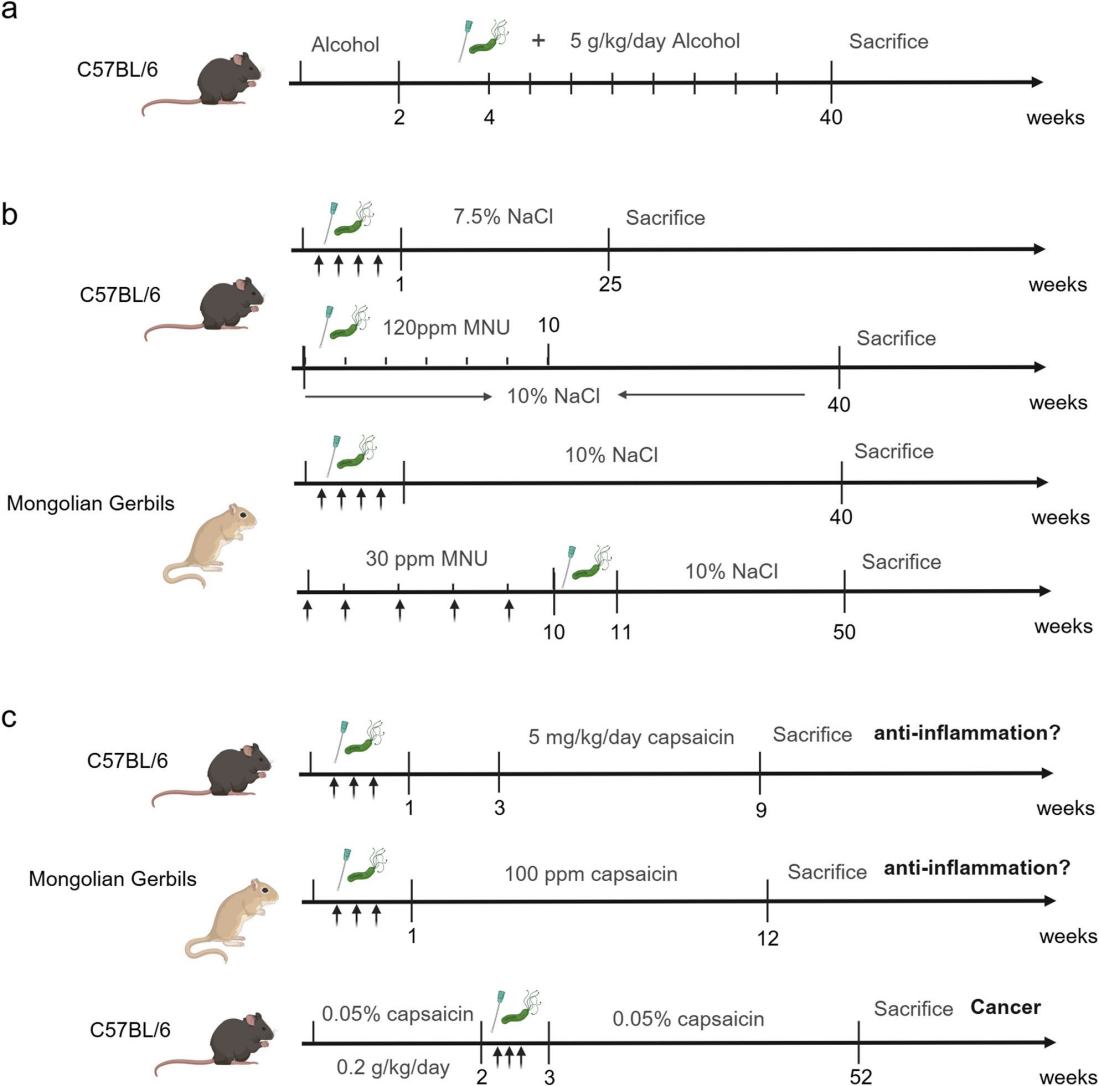
*H. pylori* combined with risk factors including alcohol, high salt, and capsaicin induced gastric cancer mouse models. **a** *H. pylori* combined with alcohol; (**b**) *H. pylori* combined with high salt; (**c**) *H. pylori* combined with capsaicin

Alcohol consumption exacerbates the effects of *H. pylori* infection, amplifying gastric mucosal injury and accelerating malignant transformation compared to either factor alone [8]. 8-week-old C57BL/6J mice first received 5 g/kg/day of alcohol for 2 weeks to prime mucosal barrier disruption. In the third week, *H. pylori* infection was initiated. To facilitate *H. pylori* colonization by reducing gastric acid given alcohol-induced fluctuations, mice were pretreated 20 mg/kg pantoprazole via oral gavage three times before receiving weekly oral gavages of *H. pylori SS1* (1 × 10⁸ CFU/mL). Alcohol exposure and *H. pylori* infection were maintained simultaneously throughout the experiment, with sequential euthanasia of 5 mice per week starting at week 4 and continuing through week 40 to track dynamic changes in gastric pathology, cytokine profiles, gene expression, and epithelial cell function [8, 96] (Fig. 4a).

The co-induced mouse model centers on the synergism between infection and lifestyle exposure, reproducing the multifactorial reality of human gastric carcinogenesis. By integrating alcohol-mediated barrier disruption, heightened oxidative stress, and metabolic toxicity with *H. pylori* virulence factors and chronic inflammation, the model elucidates the molecular circuitry through which these insults cooperate to drive malignant transformation. This model thus provides a clinically relevant platform to investigate two key translational questions: whether combining *H. pylori* eradication with alcohol cessation yields additive benefits for risk reduction, and how to target convergent pathways with multi-agent therapies.

#### *H. pylori* & High Salt

To model this clinically relevant interaction, researchers primarily use C57BL/6J mice, B6129 mice, or Mongolian gerbils, strains selected for their established susceptibility to *H. pylori* colonization and pathological progression [97–99]. The core protocol begins with *H. pylori* infection. C57BL/6J mice received intragastric inoculation of *H. pylori* (1 × 10⁹ CFU/mL, 0.1 mL) four times over 1 week to ensure stable colonization; alternatively, *H. felis* is used in some studies to induce analogous mucosal damage [100]. Following infection confirmation, mice are switched to a high-salt diet (typically 7.5%−10% NaCl, formulated in AIN76 or CE-2 basal diets) to drive synergistic pathology, while control groups receive a normal-salt diet (0.25%−0.75% NaCl) to isolate the effect of salt [97–99]. The experimental timeline ranges from 16 to 50 weeks, with sequential euthanasia at key time points (16, 24, 32, 45, 50 weeks) to track stepwise progression from chronic atrophic gastritis (CAG) and intestinal metaplasia to dysplasia and adenocarcinoma via histological staining, cytokine profiling, and molecular analysis of pro-carcinogenic pathways [98, 99, 101–103]. Besides, Mongolian gerbils also serve as a valid animal model for gastric cancer induction by *H. pylori* combined with a high-salt diet [104, 105].

Notably, some studies further refine this model by integrating a third factor (chemical carcinogens) to better recapitulate the multi-factorial nature of human gastric cancer. For example, C57BL/6J mice received alternate-week oral gavage of *H. pylori SS1* (1.0 × 10⁸ CFU) for 7 total doses, concurrent with 120 ppm MNU in drinking water for 5 cycles, while maintaining a 10% NaCl high-salt diet throughout the 40-week study [11]. This triple-factor model accelerates tumorigenesis and increases pathological severity, making it valuable for investigating convergent mechanisms of infection, diet, and chemical insult.

A critical unresolved question in these models was the impact of variable high-salt formulations. Different laboratories used NaCl concentrations ranging from 2.5% to 10%, and work by Kato et al. [106] clarified this ambiguity by demonstrating a dose-dependent effect in *H. pylori*-infected Mongolian gerbils. In their study, 6-week-old male gerbils treated with 30 ppm MNU and *H. pylori* ATCC43504 showed gastric cancer incidence rising from 15% (0.32% NaCl, normal diet) to 63% (10% NaCl diet), with intermediate rates at 2.5% and 5% NaCl. This finding confirms that salt dosage directly modulates carcinogenic synergy, underscoring the need for standardized NaCl concentrations in future studies to ensure cross-experimental reproducibility (Fig. 4b). The translational value of the *H. pylori*-high salt model lies in its alignment with epidemiological data, particularly in East Asian populations where high-salt diets and *H. pylori* prevalence coexist with elevated gastric cancer rates.

#### *H. pylori* & Capsaicin

The impact of capsaicin on *H. pylori*-induced gastritis is multifaceted, contingent upon experimental design, dosage, animal model, and research stage (Fig. 4c). This variability has long obscured its clinical relevance, making standardized murine models critical for resolving conflicting observations.

8-week-old male C57BL/6J mice first received a 7-day oral antibiotic cocktail (ciprofloxacin, metronidazole, erythromycin, albendazole) to deplete commensal gut bacteria, eliminating microbial crosstalk that could confound results. Mice were then infected with *H. pylori SS1* (1 × 10⁸ CFU/0.2 mL) via alternate-day oral gavage for 1 week, followed by a 14-day isolation period to allow for initial mucosal injury development. Subsequent daily oral administration of 5 mg/kg capsaicin for 40 days reduced *H. pylori*-induced gastric inflammation and mucosal damage, as evidenced by decreased inflammatory cell infiltration and lower histological injury scores [107]. Findings replicated in a Mongolian gerbil model, where 6-week-old males infected with *H. pylori* ATCC43504 (1 × 10⁸ CFU/mL) and fed 100 ppm capsaicin for 11 weeks showed attenuated gastritis and downregulated expression of pro-inflammatory cytokines (IL-1β, TNF-α) [108].

In contrast, a carcinogenic effect of capsaicin emerges when administered before *H. pylori* infection. 8-week-old male C57BL/6J-219 mice were pre-treated with capsaicin (0.05% or 0.2 g/kg/day) for 2 weeks, followed by infection with *H. pylori SS1* (1 × 10⁸ CFU/mL) via oral gavage three times weekly. To lower gastric acidity, mice received pantoprazole (20 mg/kg) orally three times prior to infection. Capsaicin treatment continued throughout the 52-week study. Sequential euthanasia revealed the onset of gastritis at week 32 and invasive gastric adenocarcinoma by week 52, findings that highlight capsaicin’s ability to exacerbate infection-driven malignant transformation when given prior to pathogen exposure [109]. Thus, a more comprehensive animal model is essential for elucidating the carcinogenic effects of capsaicin in the context of *H. pylori* infection.

Clinically, it guides personalized dietary recommendations. For individuals with established *H. pylori* infection, low-dose capsaicin may offer modest anti-inflammatory benefits, while high-dose or pre-infection exposure, such as in populations with habitual spicy food intake before infection diagnosis, could increase risk. However, capsaicin dosages vary widely across studies (5 mg/kg, 100 ppm, 0.05%−0.2 g/kg), with no consensus on concentrations that mimic human dietary intake, complicating cross-study comparisons. In addition, strain-specific differences in host susceptibility are underappreciated.

#### *H. pylori* & Stress

Chronic stress exerts a promotional effect at multiple stages of tumorigenesis and progression by activating the neuroendocrine and immune systems [110]. Early efforts to model this interaction date to 1999, when Matsushima et al. [111] established a foundational BALB/c mouse model to investigate *H. felis* and water-immersion stress co-induced mucosal injury. In this study, 5-week-old female mice were orally inoculated with *H. felis* (10⁹ CFU/mL, 0.8 mL) to establish infection, followed by 24-hour water immersion (20 °C) at 8 weeks post-infection to induce acute stress. Euthanasia and tissue analysis were performed immediately and 72 h post-stress to assess ulcer index and histological changes via Warthin-Starry silver staining and H&E. While this model was the first to demonstrate stress-induced augmentation of microbe-mediated gastric damage, it had notable limitations. A single large-volume gavage (0.8 mL) increased procedural variability, and the short experimental duration (72 h post-stress) failed to capture the chronicity of human gastric carcinogenesis, restricting its utility for studying long-term pathological progression.

Subsequent refinements in 2002 by Kim et al. [112] addressed these gaps by developing a chronic stress model in C57BL/6J mice. Mice received three alternate-day oral gavages of *H. pylori SS1* (1.0 × 10⁹ CFU/mL) over 5 days to ensure stable colonization; after a 12-week period to allow for initial inflammatory changes, half the mice were subjected to 12-hour water-immersion restraint stress (WIRS) three times weekly for 8 weeks (immersion in 20 °C water up to the xiphoid process). Gastric mucosal lesions were evaluated macroscopically via ulcer index (sum of erosion lengths) and microscopically using the updated Sydney system to grade inflammation and atrophy, improvements that enhanced pathological assessment reproducibility. This model’s reliance on a single stressor (WIRS) limited its ability to mimic the heterogeneous stress exposure patterns observed in humans.

**Fig. 5:**
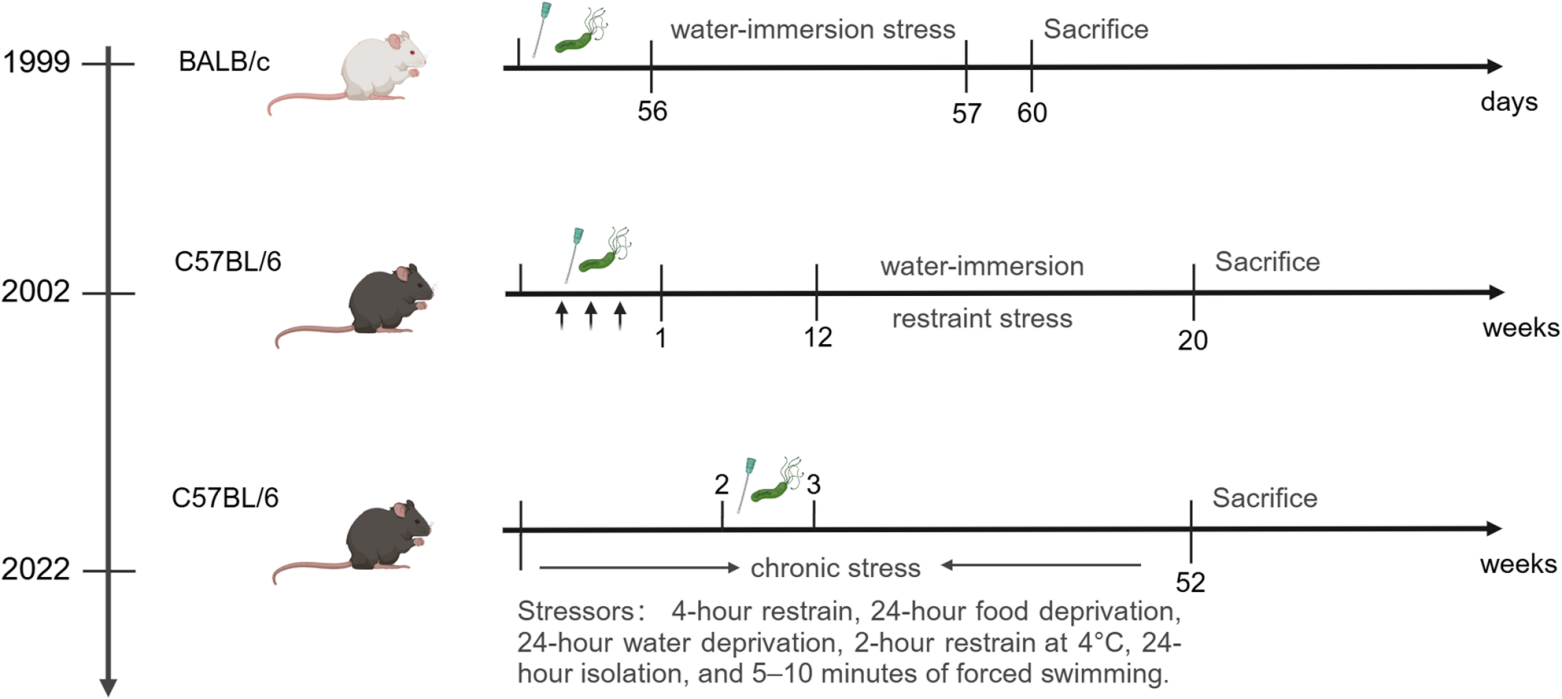
*H. pylori* combined with stress induced gastric cancer mouse models. From 1999 to 2022, researchers used different methods to construct stress-induced gastric cancer models

A more clinically relevant model was reported in 2022 by Aziz et al. [9], who integrated chronic, unpredictable stress with *H. pylori* infection to recapitulate the multifactorial nature of human gastric cancer. C57BL/6J mice first underwent 2 weeks of randomized stressors (8:00–20:00 daily) to avoid habituation, including 4-hour restraint, 24-hour food/water deprivation, 2-hour cold restraint (4 °C), 24-hour isolation, and 5–10 min of forced swimming. Mice were then infected with *H. pylori SS1* (10⁸ CFU/mL) via three oral gavages over 1 week, with pantoprazole (20 mg/kg) administered pre-infection to reduce gastric acid and facilitate colonization; chronic stress was maintained throughout the 52-week study. Sequential euthanasia of 10 mice every 4 weeks enabled dynamic tracking of pathological progression via H&E staining, revealing a stepwise transition from chronic gastritis to dysplasia and adenocarcinoma, findings that align with the protracted timeline of human infection-associated carcinogenesis (Fig. 5).

The co-induced mouse model mirrors how psychosocial factors reshape the gastric microenvironment to accelerate infection-driven malignant transformation. This platform allows testing of combined stress management and *H. pylori* eradication, discovery of early biomarkers, and screening of multi-target agents, ultimately reducing gastric cancer burden linked to infection and chronic stress. Despite their utility, stressor standardization remains a critical gap because no consensus exists on variables (stress duration, intensity, or composition) that directly impact inflammatory and tumorigenic outcomes.

#### *H. pylori* & Genetically Engineered Models

Genetically engineered mouse models (GEMMs) have transformed the study of *H. pylori*-associated gastric carcinogenesis by enabling precise dissection of how host genetic defects collude with bacterial infection to drive malignant transformation, filling a critical gap left by conventional infection models, which fail to recapitulate the genetic heterogeneity of human disease.

A foundational example is the *Tff1* (trefoil factor 1) knockout (KO) model, which interrogates the role of this mucosal protective protein in constraining *H. pylori*-induced damage. When *Tff1* KO mice are orally infected with *H. pylori SS1*, they exhibit a stark acceleration of gastric carcinogenesis compared to wild-type (WT) littermates. All *Tff1* KO mice develop gastric lesions post-infection, with 33% progressing to invasive adenocarcinoma by 32 weeks. In contrast, only 9.2% of uninfected *Tff1* KO mice develop analogous lesions, and *Tff1* WT mice remain free of proliferative or precancerous changes even after infection. This finding directly validates *Tff1* as a critical tumor suppressor that mitigates *H. pylori*-driven malignant transformation, with its loss removing a key barrier to infection-associated inflammation and epithelial dysplasia [113]. Another pivotal model explores the synergy between *H. pylori* or *H. felis* and hypergastrinemia, a condition linked to human gastric atrophy and cancer risk. *INS-GAS* transgenic mice, which overexpress gastrin under an insulin promoter, were infected with *H. felis* at 4 weeks of age, resulting in accelerated pathological progression relative to wild type (WT) FVB/N mice. By 6–7 months post-infection, 85% of *INS-GAS* mice develop mucosal carcinoma, 54% show submucosal invasion, and 46% exhibit vascular invasion, mirroring the aggressive behavior of human gastric cancer in settings of chronic hypergastrinemia [114].

Models targeting key signaling pathways have also clarified how *H. pylori* exploits host pathway defects to exacerbate carcinogenesis. For instance, *epidermal growth factor receptor* (*EGFR*) epithelial-specific knockout mice (*Egfr*Δepi) infected with *H. pylori SS1* display more severe gastric inflammation and higher levels of DNA damage than WT controls, attributing to EGFR’s role in regulating mucosal repair and inflammatory restraint. Treatment with the EGFR inhibitor gefitinib reverses these phenotypes, reducing inflammatory cell infiltration and chemokine expression, underscoring the therapeutic potential of targeting EGFR in *H. pylori*-infected individuals with pathway hyperactivation [115]. Similarly, *MMP7*^*−/−*^ mice and *Nod1*^*−/−*^ mice infected with *H. pylori* exhibit heightened gastritis and increased incidence of mucosal hyperplasia and dysplasia [74, 116].

Currently, numerous studies employ knockout mice for the target gene and then induce gastric cancer by combining the carcinogen MNU with *H. pylori* infection or by simultaneously knocking out other known critical genes like *c-MYC* to elucidate its impact on gastric cancer [117]. Transgenic mice with gastric specific overexpression of pro-inflammatory cytokine GM-CSF (*GmcsfTg*) spontaneously developed gastritis in the absence of *H. pylori* infection [118]. Future developments aim to create more clinically relevant models that better recapitulate human disease, including the use of advanced gene editing technologies like CRISPR/Cas9 [119, 120].

The *H. pylori*-GEMMs overcomes the limits of purely environmental or single-gene approaches by precisely mirroring the genetic-environmental interplay that defines human gastric carcinogenesis. It reveals how germline defects (loss-of-function tumor suppressors or inflammatory-gene polymorphisms) reshape post-infection signaling like Wnt and NF-κB, the inflammatory milieu, and mutational load, thereby accelerating malignant conversion. This platform enables the creation of models for hereditary diffuse gastric cancer and other high-risk genotypes, facilitates biomarker discovery, and guides the development of genotype-tailored therapeutics, advancing precision prevention and treatment of gastric cancer.

### *Streptococcus Anginosus* Infection Induced Gastric Cancer Mouse Model

*Streptococcus anginosus* (*S. anginosus*) has emerged as a clinically relevant non-*Helicobacter* pathogen in gastric carcinogenesis, with accumulating evidence showing its enrichment in the gastric mucosa of gastric cancer patients and its ability to colonize the murine stomach. Upon colonization, *S. anginosus* drives pathological progression from acute/chronic gastritis and parietal cell atrophy to mucinous metaplasia, ultimately promoting tumorigenesis by disrupting gastric barrier function, enhancing cell proliferation, and inhibiting apoptosis [14, 121]. Given that *S. anginosus* remains a newly identified carcinogen, most studies use *H. pylori* as a positive control to contextualize its pathogenic potential.

**Fig. 6:**
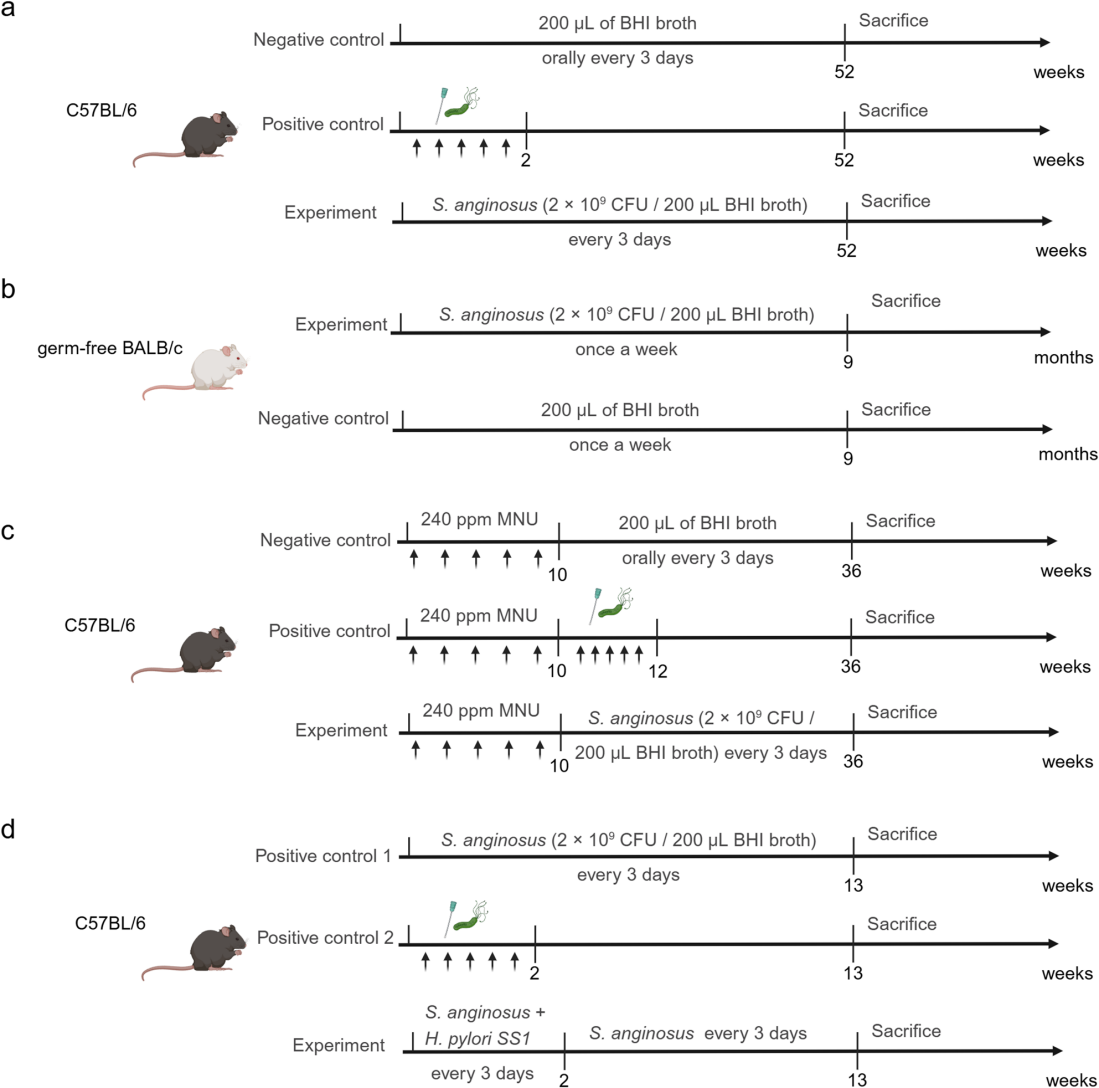
*S. anginosus* induced gastric cancer mouse models. **a** For the conventional mouse model, compared to *H. pylori*, check the function of *S. anginosus*; (**b**) For the germ-free mouse model, check the function of *S. anginosus*; (**c**) *S. anginosus* combined with MNU induced gastric cancer mouse models; (**d**) *S. anginosus* combined with *H. pylori* induced gastric cancer mouse models

The conventional *S. anginosus* model typically uses C57BL/6J mice, divided into three groups: *S. anginosus* infection, *H. pylori SS1* infection (positive control), and Brain and Heart Infusion (BHI) broth administration (negative control). Mice in the infection groups receive oral gavage of 2 × 10^9^ CFU *S. anginosus* or *H. pylori SS1* in BHI every 3 days (5 total doses for *H. pylori*, continuous dosing for *S. anginosus*), with sacrifice after 2 weeks, 3 months, 6 months, 9 months, and 12 months post-infection. Histological analysis reveals a time-dependent progression from gastritis to overt gastric cancer in *S. anginosus*-infected mice, mirroring the stepwise transformation observed in human disease and confirming *S. anginosus*’ independent carcinogenic capacity relative to *H. pylori* (Fig. 6a).

To eliminate microbiota-related confounders and isolate *S. anginosus*’ intrinsic pathogenicity, germ-free mouse models have been established using 8-week-old male BALB/c germ-free mice. These mice receive weekly oral gavage of 2 × 10^9^ CFU *S. anginosus* (or BHI) for 9 months, followed by sacrifice and gastric tissue analysis. This model confirms that *S. anginosus* alone without synergistic effects from other commensal bacteria induces significant gastric mucosal damage, validating its role as a standalone driver of gastric pathology (Fig. 6b).

For studies investigating synergistic carcinogenesis, a combined *S. anginosus* plus MNU model has been developed. Five-week-old male C57BL/6J mice first undergo five cycles of 240 ppm MNU in drinking water (alternate weeks). After MNU treatment, mice receive oral gavage of *S. anginosus*, *H. pylori SS1*, or BHI every 3 days (5 doses for *H. pylori*). Sacrifice at week 36 allows assessment of gastric cancer incidence, with preliminary data indicating that *S. anginosus* enhances MNU-driven carcinogenesis, analogous to *H. pylori* but with distinct kinetic or severity profiles that require further quantification (Fig. 6c).

However, unlike *H. pylori*, which establishes stable colonization after limited gavage doses, *S. anginosus* requires continuous or frequent gavage to maintain gastric colonization. This discrepancy may not reflect natural infection dynamics in humans. After continuous gavage of *S. anginosus* for 2 weeks (5 times), does pre-existing *S. anginosus* continue to drive gastritis-to-cancer progression? Anyway, *S. anginosus* models fill a critical gap in understanding non-*Helicobacter* microbial drivers of gastric cancer, providing a framework to investigate how emerging pathogens interact with the gastric microenvironment to promote malignancy.

### Polymicrobial Infection Induced Gastric Cancer Mouse Model

The human stomach harbors a diverse microbiome, not just single pathogens, so polymicrobial models aim to replicate this dysbiotic ecosystem, with the goal of uncovering how microbial communities rather than individual species drive carcinogenesis [122–126]. Against this backdrop, the polymicrobial infection-induced gastric cancer mouse model has become a critical tool in preclinical research. By recapitulating the complex gastric microenvironment observed in humans, this model enables the dissection of how entire microbial consortia, rather than isolated species, orchestrate oncogenic processes, providing an experimentally tractable yet clinically relevant platform to decode the holistic impact of microbiome-host interactions in gastric cancer.

An example is the co-infection model of *S. anginosus* and *H. pylori SS1* [127]. In this model, male C57BL/6J mice were divided into three groups: *S. anginosus* group, *H. pylori SS1* group, and a combined *S. anginosus* and *H. pylori SS1* group. Mice in the combined group received oral gavage of both bacteria every 3 days for 5 total doses, followed by continued *S. anginosus* gavage every 3 days; the *H. pylori* group received 5 total gavages, and the *S. anginosus* group received continuous gavages. After 3 months of infection, euthanasia and gastric tissue analysis revealed that co-infection elicited a synergistic inflammatory response with higher histological scores than either single infection, denser neutrophil/lymphocyte infiltration, and an earlier onset of the atrophy-metaplasia-dysplasia sequence, confirming a cooperative carcinogenic axis that exceeds the pathological damage of individual pathogens (Fig. 6d).

Beyond synergistic pathogens, several studies have explored microorganisms that exert antagonistic effects against *H. pylori in vivo*, offering insights into microbiota-based intervention strategies. For example, *BCF-01* effectively ameliorates *H. pylori*-induced gastric mucosal injury by curbing the pathogen’s colonization, reinstating gastric microbiota homeostasis, and interrupting the TLR4/NF-κB/pyroptosis inflammatory cascade [128]. To establish this model, 4-week-old C57BL/6 mice received a daily gavage of a triple-antibiotic cocktail (ampicillin 10 mg/mL, clarithromycin 2 mg/mL, and metronidazole 25 mg/mL; 300 µL total) for three consecutive days to eradicate the resident gastric microbiota and disrupt the mucosal barrier. Mice were then challenged with daily gavage of *H. pylori SS1* (1 × 10^10^ CFU/mL, 300 µL) for 52 days to induce chronic infection. The *BCF-01* intervention group received an additional gavage of *BCF-01* (1 × 10^10^ CFU/mL, 300 µL) 7 h after each *H. pylori* inoculation for 7 consecutive days. On day 60, euthanasia and analyses confirmed *BCF-01*’s protective effects, though the model’s reliance on antibiotic pretreatment must be noted, as this step may alter the gastric microenvironment baseline and influence subsequent microbial interactions. Another notable polymicrobial model, developed by Mariela Artola-Borán, combined *H. pylori PMSS1* with *Mycobacterium bovis BCG* (a surrogate for *M. tuberculosis*) to investigate how infection order modulates pathogen colonization, immune responses, and tissue pathology. Specifically, two principal infection sequences were tested: (i) *Mycobacterium bovis BCG* priming, wherein mice received intraperitoneal or intranasal *Mycobacterium bovis BCG* two weeks before *H. pylori* gavage, followed by five-week or three-month co-infection, and (ii) *H. pylori* priming, in which oral *H. pylori* administration two weeks before intraperitoneal *Mycobacterium bovis BCG*, followed by four-week co-infection. Single-pathogen controls (either *H. pylori* or *Mycobacterium bovis BCG* alone) and saline controls were included to compare colonization levels, frequencies of Th1/Th17/Treg cells, cytokine profiles, and the gastric/hepatic histopathological damage [129]. Moreover, nearly all published mouse models examining the impact of probiotics on *H. pylori* infection involve post-infection probiotic administration, with antibiotic pretreatment remaining optional rather than mandatory [128, 130, 131]. However, to evaluate the prophylactic efficacy of probiotics against *H. pylori* infection, the probiotics are administered first, followed by subsequent *H. pylori* challenge [131, 132].

Notably, current polymicrobial models suffer from several key limitations. Most models focus on dual-species co-infection, failing to recapitulate the multi-kingdom interactions (bacteria, fungi, viruses) that characterize the human gastric microbiome. Antibiotic pretreatment used in many models to eliminate resident microbiota and facilitate pathogen colonization may irreversibly alter the gastric mucosal microenvironment, introducing confounding variables that do not reflect natural infection dynamics. Despite these limitations, polymicrobial models fill a critical gap in understanding how microbial communities contribute to gastric carcinogenesis, complementing single-pathogen models by capturing the ecological complexity of human disease.

## Genetically Engineered Gastric Cancer Mouse Model

GEMMs have transformed gastric cancer research by enabling precise manipulation of oncogenes, tumor suppressors, and signaling pathways, addressing a critical limitation of chemical or microbial models, which often fail to recapitulate the genetic drivers of human disease. These models are built on advanced genetic tools (Cre-loxP, CRISPR/Cas9) that allow spatiotemporal control of gene editing, avoiding embryonic lethality and enabling studies of adult-onset carcinogenesis, mirroring the age-related nature of human gastric cancer [119, 133, 134]. Despite their significant contributions, GEMMs are not without limitations. For instance, species-specific differences between mice and humans can affect the translatability of research findings. Additionally, replicating the full spectrum of human gastric cancers in mouse models remains a considerable challenge, as the genetic and microenvironmental complexities of human tumors are difficult to fully recapitulate in mice [135, 136].

### *Cdh1*^loxP/loxP^ and *Trp53*^loxP/loxP^

E-cadherin (encoded by the *CDH1* gene) and p53 (encoded by the *TP53* gene) frequently exhibit genetic and epigenetic alterations in diffuse-type gastric cancer (DGC) [137, 138]. Shu Shimada et al. [139] developed a dual conditional knockout (DCKO) mouse model by crossing *Atp4b-Cre* transgenic mice with *Cdh1*^loxP/loxP^ and *Trp53*^loxP/loxP^ conditional knockout mice. In this model, E-cadherin-negative, non-polarized parietal cells were observed at 3 months of age; these cells were displaced from the gastric glands and displayed abnormal morphology. By 6 months, some DCKO mice had developed intramucosal carcinoma. At 9 months, 88% of DCKO mice exhibited intramucosal carcinoma, primarily composed of poorly differentiated cancer cells and some signet ring cells, resembling early-stage human gastric cancer. Additionally, some mice developed invasive carcinoma. By 12 months, 69% of DCKO mice had invasive cancer, with tumor cells infiltrating the submucosal and serosal layers and demonstrating high invasiveness and lymph node metastatic potential. This represents the first genetically engineered mouse model of diffuse gastric cancer, successfully recapitulating the pathological features and molecular mechanisms observed in human DGC.

### *SV40 T* and *CEA*

The *SV40 T* antigen gene, a potent oncogene, is frequently employed to induce tumor formation in transgenic mice [140]. *CEA* (carcinoembryonic antigen), a widely recognized tumor marker, is present in various adenocarcinomas, including gastric and colorectal cancers [141]. John Thompson et al. [142] constructed a transgenic mouse model (L5496) by microinjecting a *CEA* promoter/*SV40 T* antigen construct into mouse embryos. In this model, small atypical cell foci indicative of early carcinogenesis appeared within the mucosal layer of the gastric antrum by day 37. By day 50, tumors were primarily confined to the mucosa but had begun to invade the submucosal layer. Ultimately, between days 100 and 130, mice succumbed to tumor-induced pyloric obstruction, with tumors having penetrated the entire gastric wall and invaded the duodenum. This progression closely mimics the development of human gastric cancer, making the L5496 model ideal for studying early intervention and treatment strategies. Notably, this model represents the first transgenic line to specifically develop invasive gastric antral carcinomas, with 100% tumor incidence and early onset. Its high concordance with human gastric cancer development makes it a valuable tool for elucidating the mechanisms underlying gastric cancer and exploring preventive measures.

### *SLC26A9*

Xuemei Liu et al. [143] investigated the morphological and molecular changes in gastric mucosa using both complete knockout mice (*slc26a9*^*−/−*^) and conditional knockout mice *(slc26a9*^fl/fl^*/Atp4b-Cre*) with specific deletion of the solute carrier family 26 member 9 (*SLC26A9*) gene in parietal cells. In *slc26a9*^*−/−*^ mice, parietal cell loss was first observed at 1 month of age. By 2 months, these mice exhibited gastric atrophy accompanied by glandular dilation. At 6 months, the gastric mucosa showed mucinous cell metaplasia, including spasmodic peptide-expressing metaplasia (SPEM) and intestinal metaplasia (IM). At 14 months, all *slc26a9*^*−/−*^ mice developed severe pre-malignant gastric lesions, including chronic atrophic gastritis (CAG), mucinous metaplasia, deep cystic formation, and high-grade intraepithelial neoplasia (HGIN). By 18 months, 4 out of 13 mice developed moderately differentiated gastric cancer, while 9 out of 13 exhibited HGIN. Similarly, in *slc26a9*^fl/fl^/*Atp4b-Cre* mice with parietal cell-specific deletion, the gastric mucosa showed progressive pathological changes. Parietal cell loss and gastric atrophy were observed at 1 month of age. Mucinous cell metaplasia occurred by 6 months, and HGIN was detected at 14 months. By 18 months, undifferentiated gastric cancer was observed, accompanied by the loss of MUC5AC and Claudin 18.2 expression. These findings demonstrate that deletion of the *SLC26A9* gene, whether complete or restricted to parietal cells, leads to a gradual deterioration of the gastric mucosa in mice, ultimately progressing to gastric cancer. This process mimics the multi-step development of human gastric cancer, transitioning from chronic atrophic gastritis to metaplasia, high-grade intraepithelial neoplasia, and ultimately to gastric cancer.

### *Apc* Δ750

Sarp Uzun et al. [144] successfully constructed a mouse model carrying the *Apc* Δ750 frameshift mutation by targeting the 16th exon of the mouse *Apc* gene using CRISPR/Cas9 technology. This mutation causes the *Apc* protein to be truncated in the seventh armadillo (ARM) domain, mimicking the characteristics of human *APC* gene mutations. The research team evaluated the phenotypic effects of the *Apc* Δ750 allele by backcrossing it from the FVB/N strain to the C57BL/6J strain in mice of different generations. The results showed that, F1-B6; FVB *Apc* Δ750 mixed background mice formed gastric polyps similar to human pyloric adenoma (PGA) in the pylorus and antrum regions of the stomach. These polyps were histologically similar to human PGA, exhibiting adenomatous hyperplasia and nuclear accumulation of β-catenin. This model not only provides a new tool for studying the pathogenesis of gastric cancer but also provides potential application value for developing therapeutic strategies and early diagnostic markers for WNT signaling pathway.

### *Muc6*^*−/−*^

Gastric cancer development is closely associated with the loss of gastric mucin, particularly MUC6 [145]. *Muc6* knockout mice (*Muc6*^*−/−*^) begin to exhibit gastric mucosal hyperplasia at 3 months of age, predominantly in the antrum region. Over time, there is significant infiltration of inflammatory cells into gastric mucosa, along with a marked increase in the number of Ki67-positive proliferating cells. Concurrently, the expression of intestinal metaplasia markers, such as CD44v and TFF2, is upregulated, indicating a gradual transformation of gastric mucosal cells into intestinal epithelial cells. By 1 year of age, all *Muc6*^*−/−*^ mice develop invasive gastric cancer, with tumor cells breaching the mucosal layer and invading the submucosal layer. During this process, the Golgi stress response leads to upregulation of GOLPH3, activation of the MAPK pathway, and subsequent promotion of cell proliferation and tumor growth. Moreover, *Muc6* deficiency results in abnormal expression of N-linked glycan chains rich in mannose, which bind to clusterin precursors and further contribute to tumor initiation and progression [146].

### *TFF1*

Trefoil factor 1 (TFF1) is a small secreted protein expressed in the surface cells of the gastric mucosa, primarily responsible for maintaining mucosal integrity and promoting repair following injury [147, 148]. In *TFF1* gene knockout (*TFF1*^*−/−*^) mice, gastric mucosal inflammation and barrier dysfunction emerge 2–4 months after birth. By approximately 6 months of age, these mice begin to exhibit low-grade dysplasia (LGD). Over time, some mice progress to high-grade dysplasia (HGD) and adenocarcinoma by around 10 months [149, 150]. This progression is characterized by aberrant activation of the IL6-STAT3 signaling pathway, which drives cell proliferation, exacerbates inflammatory responses, and contributes to the formation of a tumor microenvironment [150].

Furthermore, under MNU treatment, the progression of gastric cancer in *Tff1*-deficient (*Tff1*^*+/−*^) mice was significantly accelerated, with increased malignancy compared to wild-type mice. Specifically, *Tff1* gene expression rapidly declined and was almost completely lost following MNU treatment. Concurrently, cell proliferation and progenitor cell expansion in the gastric antrum were significantly enhanced, as evidenced by a marked increase in the proportion of Ki67- and Dclk1-positive cells. Furthermore, *Tff1*^*+/−*^ mice developed microadenocarcinomas 18 weeks after MNU treatment. By 36 weeks, tumor incidence and malignancy were significantly higher in *Tff1*^*+/−*^ mice than in wild-type mice, with tumors primarily manifesting as mucosal adenocarcinomas and invasive adenocarcinomas. These changes are closely associated with epigenetic alterations in the *Tff1* promoter region, including DNA methylation and histone H3K9 methylation. These findings indicate that a haploinsufficiency of the *Tff1* gene significantly increases the risk of gastric cancer [16].

By mating *LoxP-Top-LoxP* (*LSL*) - *hMYH9* transgenic mice with *Atp4b-cre* mice, transgenic mice with gastric parietal cell specific overexpression of MYH9 were generated; By mating *Myh9* conditional knockout mice (*Myh9*^fl/fl^) with *Atp4b-cre* mice, transgenic mice with gastric parietal cell specific knockout of *Myh9* were generated. Mating transgenic mice overexpressing or knocking out *MYH9* with *Tff1* gene knockout mice (*Tff1*^*−/−*^) to generate composite transgenic mice. In *LSL-hMYH9*; *Atp4b-cre*; *Tff1*^*−/−*^ mice, the incidence of tumors and the tumor volume significantly increased; In *Myh9*^fl/fl^; *Atp4b-cre*; *Tff1*^*−/−*^ mice, the incidence of tumors is significantly reduced and the tumor volume decreases [151].

### *INS-GAS*

Insulin-Gastrin (*INS-GAS*) transgenic mice are genetically modified mice with the human gastrin gene inserted into their genome, which is controlled by an insulin promoter for expression [152]. This design allows mice to simulate the pathological state of hypergastrinemia, where gastrin levels are elevated. Select wild-type FVB/N mice with the same genetic background as *INS-GAS* mice as the control group to eliminate the interference of genetic background on the experimental results. The experiment is divided into short-term (1–4 months) and long-term (5–20 months) observation stages to evaluate the acute and chronic effects of hypergastrinemia on gastric mucosa. At different time points (such as 1 month, 4 months, 12 months, 20 months), mice were euthanized and gastric tissue samples were collected for subsequent analysis. The results demonstrated that *INS-GAS* mice exhibited increased gastric acid secretion and parietal cell counts during the early phase (1–4 months). However, as time progressed (beyond 5 months), both gastric acid secretion and parietal cells gradually declined, ultimately leading to gastric atrophy. By 20 months, *INS-GAS* mice showed significant gastric mucosal thickening, intestinal metaplasia, dysplasia, and carcinoma in situ, with 6/8 of *INS-GAS* mice developing invasive gastric cancer with vascular invasion [114]. In addition, based on this, the influence of other oncogenes on the development of gastric cancer can also be studied. For example, Krakowiak et al. [74] utilized the *INS-GAS* mouse model to explore the impact of *MMP7* deficiency on *H. pylori*-induced gastric precancerous lesions. In their experiment, both wild-type (WT) and *MMP7* knockout (*MMP7*^*−/−*^) *INS-GAS* mice were challenged with *H. pylori SS1* or treated with broth alone as a control. After 12 weeks, the researchers performed pathological assessments on the gastric tissues of the mice, focusing on the degree of inflammation, incidence of mucosal hyperplasia, and dysplasia (precancerous lesions). The findings revealed that *MMP7*^*−/−*^ mice exhibited significantly more severe gastric inflammation and higher incidences of mucosal hyperplasia and dysplasia following *H. pylori* infection. These results indicate that *MMP7* deficiency also exacerbates the development of precancerous lesions in the stomach, potentially contributing to gastric carcinogenesis. Giovanni Suarez et al. [116] utilized the *INS-GAS* mouse model to explore the impact of *NOD1* deficiency on gastric inflammation and precancerous lesions induced by *H. pylori*. In their study, both wild-type (*Nod1*^*+/+*^) and *NOD1* knockout (*Nod1*^*−/−*^) *INS-GAS* mice were infected with the *H. pylori SS1* or treated with broth alone as controls. The gastric tissues of these mice were evaluated pathologically at 2, 20, 40, and 90 days after infection, with assessments focusing on inflammation severity, mucosal hyperplasia, and dysplasia (precancerous lesions). The findings revealed that *Nod1*^*−/−*^ mice developed more severe gastric inflammation following infection. Moreover, the incidence of gastric dysplasia was significantly higher in *Nod1*^*−/−*^ mice compared to *Nod1*^*+/+*^ mice at both 40 and 90 days after infection. Notably, even uninfected *Nod1*^*−/−*^ mice exhibited elevated levels of inflammation and tissue damage at 40 and 90 days. These results collectively suggest that *NOD1* deficiency exacerbates *H. pylori*-induced gastric inflammation and accelerates the development of precancerous lesions.

As mentioned earlier, a high salt diet is also a risk factor for gastric cancer, and the combination of *H. pylori* and high salt induces the occurrence of gastric cancer. So, what is the effect of the combination of *H. pylori* infection and high salt on the occurrence of gastric cancer in *INS-GAS* transgenic mice? How can we construct the mouse model? James G. Fox et al. [153] employed 86 *INS-GAS* transgenic mice (43 males and 43 females) to investigate the effects of *H. pylori* infection and dietary salt intake on gastric pathology. The mice were divided into two primary groups: one infected with *H. pylori* and the other remaining uninfected. Each group was further subdivided into two dietary regimens: a high-salt diet (7.5%) and a basal diet (0.25%). Infection with *H. pylori* was achieved via oral administration of *H. pylori SS1*. Anatomical and histopathological analyses were performed at 5 and 7 months after infection to assess the progression of gastric lesions. The results demonstrated that *H. pylori* infection significantly exacerbated gastric pathology, including inflammation, glandular atrophy, hyperplasia, metaplasia, and dysplasia/carcinogenesis. Notably, these lesions were more severe in male mice compared to females [154]. Interestingly, a high-salt diet did not promote the development of gastric cancer in this model. Instead, it appeared to exert a protective effect, particularly in male mice infected with *H. pylori*. The impact of a high-salt diet on gastric cancer development is likely influenced by both the presence of infection and host-specific factors. Therefore, when investigating this aspect, careful consideration should be given to the construction and design of mouse models used in such studies.

Can chemical carcinogen promote the progression of gastric cancer in *INS-GAS* mice? What are the methods used by researchers? Hiroyuki Tomita et al. [16] investigated the impact of MNU treatment on gastric cancer development in *INS-GAS* mice. Both *INS-GAS* mice and wild-type (WT) mice were administered MNU via drinking water over five consecutive 1-week courses to induce gastric cancer. Following MNU treatment, the mice were observed continuously for up to 36 weeks to assess the progression of gastric cancer. The results revealed that the incidence, number, and size of antral tumors in *INS-GAS* mice were significantly lower than those in WT mice following MNU treatment. This finding indicates that hypergastrinemia significantly inhibits the development and progression of gastric cancer.

### *gp130*^F/F^

In addition, the *gp130*^757F/F^ mouse model can quickly and completely simulate the occurrence and development of gastric cancer, providing a powerful tool for studying the molecular mechanisms of gastric cancer. This type of mouse introduced a mutation in the *gp130* gene through gene knock in technology, resulting in the deletion of the SHP2 binding site on the IL-6 family receptor gp130, thereby enhancing the activity of the STAT3 signaling pathway [155]. The study started at 4 weeks of age in mice and conducted histological and immunohistochemical analysis every few weeks to monitor the occurrence and development of gastric cancer. The results showed that mice began to develop gastric antral adenomas at 4 weeks of age, accompanied by inflammation and ulcers. At 20 weeks of age, the adenoma reaches its maximum size and begins to expand to the gastric body. After 30 weeks of age, progressive changes such as gastritis, atrophy, intestinal metaplasia, dysplasia, and submucosal infiltration occur [156, 157].

### *gp130*^F/F^/*Tff2*^*−/−*^

After 12 months of chronic infection with *H. pylori*, the *Tff2* promoter undergoes methylation and leads to its silencing. *Tff2* heterozygous mice were hybridized with *gp130*^F/F^ mice to generate *gp130*^F/F^/*Tff2*^*−/−*^ compound mutant mice with *Tff2* gene knockout and compared with the *gp130*^F/F^ control group. The results showed that *Tff2* gene knockout mice exhibited larger gastric antral tumors than the control group at 6 weeks, and the tumors further enlarged at 12 weeks. This accelerated tumor growth is attributed to increased cell proliferation and loss of gastric tumor suppressor genes, highlighting the crucial role of *Tff2* in inhibiting the development of gastric cancer [158].

### *Lats1/2i*ΔLgr5

The Hippo signaling pathway, also known as the Hippo-YAP/TAZ pathway, is a critical regulator of growth control and tumor suppression, implicated in processes such as carcinogenesis, tissue regeneration, and metabolism [159, 160]. When the Hippo pathway is activated, LATS1/2 phosphorylates YAP/TAZ, thereby inhibiting their activity. Conversely, when the pathway is inactivated, YAP/TAZ accumulates in the nucleus, where it drives gene expression by forming complexes with transcription factors, particularly TEAD, leading to increased cell proliferation and inhibition of apoptosis [161]. Wonyoung Choi et al. [162] conditionally knocked out the *Lats1* and *Lats2* genes, which encode the direct negative regulators of YAP/TAZ to activate the YAP/TAZ signaling pathway. They generated *Lats1*^fl/fl^; *Lats2*^fl/fl^; *Lgr5-Cre*ER mice (referred to as *Lats1/2i*ΔLgr5 mice) by crossing floxed *Lats1* and *Lats2* mice with *Lgr5-EGFP-IRES-CreERT2* mice. In these mice, YAP/TAZ was specifically activated in *Lgr5*^+^ gastric epithelial stem cells through tamoxifen-induced Cre expression. This *Lats1/2i*ΔLgr5 mouse model revealed a progressive pathological sequence from YAP/TAZ activation to gastric cancer development. At 8 weeks post-induction, slight glandular distortion and changes in nuclear size were observed in the gastric antrum, indicative of hyperplasia. By 12 weeks, loss of nuclear polarity and low-grade intraepithelial neoplasia were evident. At 16 weeks, the glandular architecture became disordered, with significant cellular atypia, progressing to high-grade intraepithelial neoplasia. By 20 and 24 weeks, nuclei were deeply stained, glandular buds had invaded the lamina propria, and intramucosal carcinoma had developed. These findings highlight the pivotal role of YAP/TAZ activation in gastric cancer progression and provide a robust model for investigating early interventions in gastric cancer.

### *H*^*+*^*/K*^*+*^*ATPase-Cre*; *LKB1*^L/L^; *PTEN*^L/L^

Liver kinase B1 (*LKB1*) and phosphatase and tensin homolog (*PTEN*) are two critical tumor suppressor genes that inhibit tumor development by regulating the AMPK signaling pathway and the PI3K/AKT/mTOR signaling pathway, respectively [163, 164]. Kuan-Te Fang et al. [165] simulated the occurrence and progression of human gastric cancer by specifically knocking out the *LKB1* and *PTEN* genes in the stomach of mice, thereby elucidating their roles in gastric cancer development. To achieve this, they constructed an *H*^*+*^*/K*^*+*^*ATPase-Cre* transgenic mouse model capable of specifically expressing Cre recombinase in gastric parietal cells, enabling targeted knockout of the *LKB1* and *PTEN* genes. By crossing conditional knockout mice for *LKB1* and *PTEN* with *H*^*+*^*/K*^*+*^*ATPase-Cre* transgenic mice, they generated a composite knockout mouse model: *H*^*+*^*/K*^*+*^*ATPase-Cre*; *LKB1*^L/L^; *PTEN*^L/L^. Their findings revealed that the survival time of *H*^*+*^*/K*^*+*^*ATPase-Cre*; *LKB1*^L/L^; *PTEN*^L/L^ composite knockout mice were significantly reduced, with an average survival of less than 40 weeks. In contrast, control mice (*H*^*+*^*/K*^*+*^*ATPase-Cre*; *LKB1*^L/L^ and *H*^*+*^*/K*^*+*^*ATPase-Cre*; *PTEN*^L/L^) survived for over 65 weeks. Additionally, composite knockout mice began to exhibit weight loss after 20 weeks. MRI and histological analyses showed that these mice developed significantly enlarged stomachs with thickened gastric walls, submucosal hemorrhage, and multiple hyperplastic polyps in the gastric glands. Notably, approximately 68% of the composite knockout mice exhibited tumor invasion into the duodenum. Further investigation revealed increased expression of cell proliferation markers (Ki67), epithelial-mesenchymal transition (EMT) markers (such as α-SMA, vimentin, and MMP9), and stem cell markers (including CD44, CD133, c-kit, and LGR5) in the gastric tissues of composite knockout mice. These findings indicate uncontrolled gastric cell proliferation, enhanced inflammatory response, activation of the EMT process, and acquisition of stem cell characteristics. These collective changes are likely to contribute to the aggressive gastric cancer phenotype observed in this model.

### *K19-Wnt1/C2mE*

The Wnt signaling pathway and the prostaglandin E2 (PGE2) pathway are both implicated in the development of gastric cancer. Activation of the Wnt pathway can lead to abnormal cell proliferation and differentiation, while activation of the PGE2 pathway is closely associated with inflammation and tumorigenesis. To investigate the roles of these pathways in gastric cancer, Hiroko Oshima et al. [166] constructed transgenic mouse models to simultaneously activate the Wnt and PGE2 pathways. First, they generated *K19-Wnt1* transgenic mice, in which Wnt1 was specifically expressed in the gastric mucosa under the control of the Keratin 19 (*K19*) promoter. They also developed *K19-C2mE* transgenic mice, which expressed cyclooxygenase-2 (COX-2) and microsomal prostaglandin E synthase 1 (mPGES-1) in the gastric mucosa, resulting in elevated levels of PGE2. By crossing these two lines, they obtained *K19-Wnt1/C2mE* composite transgenic mice to study the synergistic effects of the Wnt and PGE2 pathways. In the *K19-Wnt1* mice, small preneoplastic lesions appeared in the gastric mucosa, characterized by the proliferation of undifferentiated epithelial cells and accumulation of β-catenin. However, in the *K19-Wnt1/C2mE* composite transgenic mice, significant gastric cancer developed by 20 weeks of age. The tumor cells exhibited nuclear atypia, irregular glandular structures, and nuclear accumulation of β-catenin. Additionally, these mice showed a marked increase in macrophage infiltration and angiogenesis within the tumor tissue. These findings demonstrate that the simultaneous activation of the Wnt and PGE2 pathways can drive the development of gastric cancer through a metaplasia-carcinoma sequence. The *K19-Wnt1/C2mE* composite transgenic mouse model provides a valuable tool for studying the molecular mechanisms underlying gastric cancer and for developing targeted therapeutic strategies.

Besides, the *HKα*^−/−^ mice, which have had the proton pump α subunit (*H*^*+*^*/K*^*+*^*-ATPase α*) gene knocked out, exhibit gastrin-dependent gastric body hyperplasia, with significant hyperplasia observed at 12 weeks of age. The *K19-C2mE* mice, engineered to overexpress cyclooxygenase-2 (COX-2) and microsomal prostaglandin E synthase 1 (mPGES-1) through transgenic technology, develop gastric body tumors, which become evident at 40 to 50 weeks of age (at 48 weeks of age) [167, 168]. Gan mice additionally express Wnt1 on this basis, leading to the occurrence of gastric tumors. The mechanism is the simultaneous activation of the Wnt signaling pathway and the COX-2/PGE2 pathway [167]. Additionally, the *K19-Wnt1/C2mE* mice, which induce atypical gastric hyperplasia by simultaneously activating the Wnt signaling pathway and the prostaglandin E2 pathway, show noticeable tumor development at 30 weeks of age [168].

### *Kras*

The Kirsten rat sarcoma viral oncogene homolog (*Kras*) gene is a well-known oncogene, and its activation through mutations is commonly observed in various cancers [169]. The Kras protein functions as a molecular switch, cycling between an active GTP-bound state and an inactive GDP-bound state via its GTPase activity [170]. *Kras* mutations result in its sustained activation, thereby driving tumorigenesis [171]. *Kras* activation is a key factor in the development of gastric cancer [172]. Yoonkyung Won et al. [173] have reviewed a variety of genetically engineered mouse models that are used to study *Kras* activation. These models simulate the occurrence and progression of human gastric cancer by specifically activating *Kras* in defined cell or tissue types. However, evidence suggests that *Kras* activation alone is insufficient to trigger the complete process of gastric cancer development [174]. Instead, *Kras* activation may require synergistic interactions with other genetic alterations, such as mutations in *Trp53*, *Cdh1*, *PTEN*, and other genes, to fully promote gastric cancer formation.

To investigate the mechanisms underlying gastric adenocarcinoma development and metastasis, Jacob E. Till et al. [175] constructed two genetically engineered mouse models: Tcon (triple conditional knockout) and Dcon-Ecad het (dual conditional E-cadherin heterozygous). Tcon mice were generated by mating *Atp4b-Cre* transgenic mice with *Cdh1*^fl/fl^, *Trp53*^fl/fl^, *LSL-Kras*^G12D^, and *Rosa26LSL-YFP* strains, thereby specifically expressing oncogenic Kras and knocking out E-cadherin and p53 in the gastric parietal cell lineage. These mice exhibited high-grade dysplasia by 3 weeks of age, with 40% developing invasive cancer by 6 weeks and 100% by 9 weeks. Tumors in Tcon mice also metastasized widely to lymph nodes, lungs, and liver. In contrast, Dcon-Ecad het mice, which retained a wild-type *Cdh1* allele, had a significantly prolonged median survival of 123 days. Only 20% of these mice developed gastric cancer, and no distant organ metastasis was observed. These findings highlight the critical role of E-cadherin in inhibiting gastric cancer development and progression, underscoring its function as a “gatekeeper” in this disease.

Daisuke Douchi et al. [176] developed a series of conditional mutation mouse models by crossing *Pgc-mCherry-IRES-CreERT2* (*Pgc-CreERT2*) mice with *Kras*^G12D^, *Apc*^flox/flox^, and *Trp53*^flox/flox^ mice. These models were designed to simulate the multi-step progression of gastric cancer. Specifically, *Pgc-CreERT2*; *Kras*^G12D/+^ mice exhibited pseudopyloric metaplasia within 3 months after induction. The addition of *Apc* inactivation (*Pgc-CreERT2*; *Kras*^G12D/+^; *Apc*^flox/flox^) led to the development of intramucosal carcinoma (IDC) characterized by irregular glandular structures and high-grade dysplastic features at 9 months post-induction. Further inactivation of *Trp53* (*Pgc-CreERT2*; *Kras*^G12D/+^; *Apc*^flox/flox^; *Trp53*^flox/flox^) resulted in highly invasive and metastatic gastric cancer, also observed at 9 months post-induction. In summary, the study successfully recapitulated the multi-step progression of gastric cancer, from metaplasia to intramucosal carcinoma, and ultimately to invasive and metastatic disease, through the sequential activation of *Kras*^G12D^ and inactivation of *Apc* and *Trp53*.

### *ARID1A*

Gastric cancer (GC) ranks as the third leading cause of cancer-related mortality globally, with frequent mutations in chromatin-modifying factor genes, particularly the *ARID1A* gene, being a notable feature [177–179]. ARID1A, a key subunit of the BAF chromatin remodeling complex, has been shown to exert tumor-suppressive effects in various cancers, but its specific role in GC remains unclear [172]. To elucidate the role of ARID1A in GC development, Adrian Kwan Ho Loe et al. [180] constructed two genetically engineered mouse models. By mating *Atp4b-Cre* transgenic mice with *Arid1a* conditional knockout (fl/fl) and Notch signaling pathway activation (*Rosa26NICD*) mice, they generated GC models with *Arid1a* heterozygosity (*Arid1a*^fl/+^) and complete *Arid1a* knockout (*Arid1a*^fl/fl^). These models specifically targeted the deletion of the *Arid1a* gene in gastric parietal cells, thereby simulating the *ARID1A* mutation scenario observed in human GC. Their findings revealed that GC in *Arid1a* heterozygous mice exhibited accelerated tumor growth and progression, increased tumor cell proliferation, and downregulation of p53 and apoptosis pathway genes. Conversely, in tumors from *Arid1a* knockout mice, the p53 pathway was aberrantly activated, leading to increased tumor cell apoptosis and a competitive disadvantage. These results highlight the dose-dependent effects of *ARID1A* in GC and provide a model basis for subsequent combination therapy strategies.

In all, genetically engineered mouse models are indispensable in gastric cancer research, offering insights into disease mechanisms and aiding in the development of new treatments. While challenges remain, ongoing advancements in genetic engineering hold promises for creating more accurate and predictive models for preclinical studies (Table 2). Genetically engineered gastric cancer mouse models, sculpted by precise allelic alterations, mirror the human paradigm of mutation-driven tumorigenesis. They illuminate how tumor-suppressor loss, oncogene activation, and pathway crosstalk orchestrate malignant evolution, while providing an indispensable platform for modeling hereditary forms, screening targeted agents, and evaluating gene therapies, accelerating the shift from empirical to precision oncology in gastric cancer.

**Table 2:** Tumor incidence and disease progression timelines in gastric cancer-related genetically engineered mouse models

Gene(s)	Tumor incidence	Disease progression and timeline
*Cdh1*^*loxP/loxP*^; *Trp53*^*loxP/loxP*^ (DCKO)	88% (intramucosal carcinoma at 9 months), 69% (invasive cancer at 12 months)	3 months: Non-polarized parietal cells 6 months: Intramucosal carcinoma 9 months: Invasive cancer 12 months: Lymph node metastasis
*SV40 T* ^*+*^ *CEA* (L5496)	100%	Day 37: Atypical cell foci in gastric antrum mucosa Day 50: Submucosal invasion Days 100–130: Full-wall invasion
*SLC26A9* ^*−/−*^	4/13 (moderately differentiated cancer at 18 months), 9/13 (HGIN at 18 months)	1 month: Parietal cell loss 6 months: Gastric atrophy, mucinous metaplasia 14 months: HGIN 18 months: Undifferentiated cancer
*Apc* Δ750	Forms pyloric adenoma-like polyps (incidence unspecified)	Mixed FVB/N-C57BL/6J background: PGA-like polyps in pylorus/antrum (β-catenin nuclear accumulation)
*Muc6* ^*−/−*^	100% (invasive cancer at 1 year)	3 months: Antral mucosal hyperplasia 1 year: Invasive cancer with MAPK activation
*TFF1* ^*−/−*^	100% lesion incidence post-H. pylori, 33% adenocarcinoma at 32 weeks	2–4 months: Inflammation 6 months: LGD 10 months: HGD/adenocarcinoma
*INS-GAS*	6/8 (invasive cancer at 20 months)	5 months: Gastric atrophy 20 months: Intestinal metaplasia, carcinoma in situ
*gp130* ^*F/F*^	Progressive lesions starting at 4 weeks	4 weeks: Antral adenoma 20 weeks: Tumor expansion 30 weeks: Submucosal invasion
*gp130* ^*F/F*^ */Tff2* ^*−/−*^	Accelerated tumor growth vs. controls	6 weeks: Larger antral tumors 12 weeks: Tumor enlargement with increased proliferation
*Lats1/2i*ΔLgr5	Intramucosal carcinoma at 20–24 weeks	8 weeks: Glandular distortion 12 weeks: Low-grade neoplasia 16 weeks: High-grade neoplasia 20–24 weeks: Intramucosal carcinoma
*H*^*+*^*/K*^*+*^ *ATPase-Cre; LKB1*^*L/L*^; *PTEN*^*L/L*^	68% duodenal invasion; < 40 weeks survival	20 weeks: Gastric wall thickening, polyps < 40 weeks: EMT activation, stem cell marker upregulation
*K19-Wnt1/C2mE*	Significant cancer at 20 weeks	20 weeks: Nuclear atypia, irregular glands (β-catenin accumulation)
*Kras* (Tcon model)	100% invasive cancer at 9 weeks	3 weeks: High-grade dysplasia 6 weeks: 40% invasive cancer 9 weeks: 100% invasive cancer with metastasis
*ARID1A* ^*fl/+*^	Accelerated tumor progression	Heterozygous: p53 pathway suppression Homozygous: p53 activation, apoptosis (time unspecified)
*LSL-hMYH9; Atp4b-cre; Tff1* ^*−/−*^	Increased tumor incidence/volume	*MYH9* overexpression + *TFF1*^−/−^: Enhanced tumorigenesis *MYH9* knockout + *TFF1*^−/−^: Reduced tumors (time unspecified)

*HGIN* High-grade intraepithelial neoplasia, *LGD/HGD* Low/High-grade dysplasia, *EMT* Epithelial-mesenchymal transition, *PGA* Pyloric gland adenoma

## Xenograft Gastric Cancer Mouse Models

Xenograft gastric cancer mouse models, encompassing cell-derived xenografts (CDX) and patient-derived xenografts (PDX), occupy a unique niche in preclinical research by directly leveraging human tumor materials, complementing the mechanistic insights of chemical carcinogen, microbial, and genetically engineered models. Unlike those models, which recapitulate etiologies of gastric cancer (environmental chemicals, *H. pylori* infection, genetic mutations), xenograft models prioritize the preservation of human tumor biology, a critical advantage for translating findings to clinical practice.

### Cell Derived Xenograft Mouse Model

CDX models are established by inoculating well-characterized human gastric cancer cell lines into immunodeficient mice, leveraging the genetic stability of cell lines to enable reproducible, low-cost, and rapid in vivo studies. Their primary utility lies in exploring cell-autonomous mechanisms (such as oncogene-driven proliferation and drug resistance) and conducting large-scale preliminary drug screens, addressing a key limitation of GEMMs (which require months to develop tumors) and PDXs (which are resource-intensive). Critical to model validity is the precise matching of cell lines, recipient mice, and inoculation methods to research objectives. Common gastric cancer cell lines include MKN-45 (poorly differentiated, diffuse-type, for metastasis studies), AGS (wild-type TP53, for signaling pathway analysis), and YTN16 (scirrhous-type, for peritoneal dissemination models) and so on, each selected based on the biological question [151, 181]. Recipient mice are stratified by immunodeficiency level. BALB/c NU/NU mice (thymus-deficient, moderate immunodeficiency) are cost-effective for basic proliferation assays; NSG mice (NOD-SCID-IL2Rγ⁻/⁻, severe immunodeficiency) exhibit superior human cell engraftment and are preferred for long-term (≥ 8 weeks) studies or models requiring high tumor fidelity; SCID mice remain useful for peritoneal metastasis models due to their tolerance for intraperitoneal cell injection [182, 183].

**Fig. 7:**
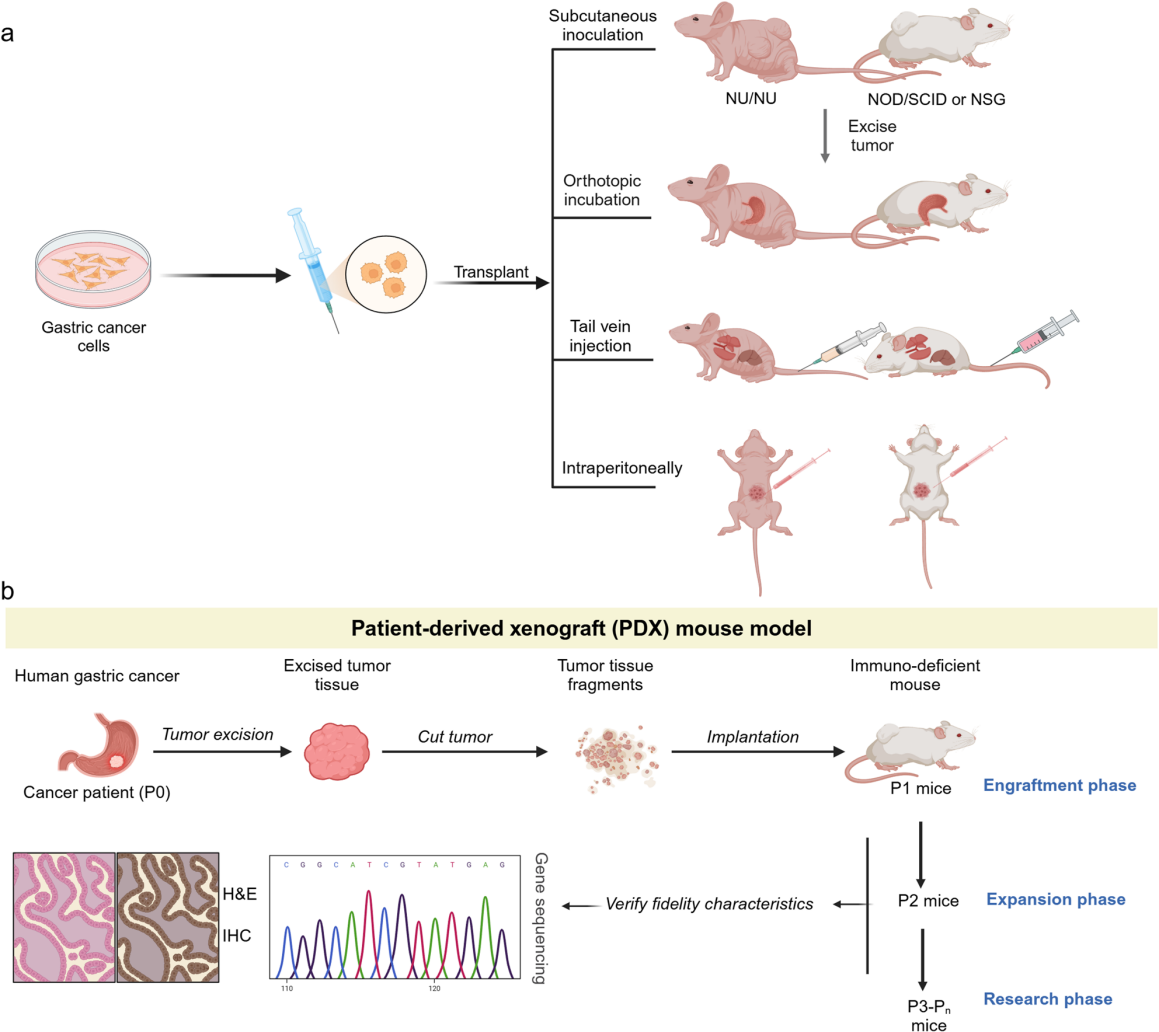
The construction of patient derived xenograft mouse model and cell derived xenograft mouse models. **a** Cell derived xenograft mouse models include subcutaneous transplant tumor model, orthotopic transplant tumor model, and metastasis related models (tail vein injection and intraperitoneal injection). Cultivate enough gastric cancer cells, digest them into individual cells, and then transplant the cells to specific sites according to the experimental purpose. Regularly observe and record the growth of tumors. **b** After obtaining the consent of the gastric cancer patient, tumor tissue was taken and transported on ice. The tumor tissue was cut into small pieces and transplanted subcutaneously into immunodeficient mice (P1). After the tumor grows, the tumor tissue is removed, cut into small pieces, and then transplanted subcutaneously into other immunodeficient mice, and these steps can be repeated (P2-Pn). In the expansion phase, it is necessary to check whether the heritability of tumor tissue has changed through H&E, IHC or cell sequencing

Subcutaneous inoculation is the most widely used approach, involving injection of 1 × 10⁶−8 × 10⁶ viable cells (suspended in 50% Matrigel to enhance adherence) into the dorsal flank of 6–8-week-old mice. Tumors typically form within 2–3 weeks, and mice are randomized to treatment groups once tumor volumes reach 100 mm³ (calculated via V = 0.5*long axis*short axis^2). This method is operationally simple but rarely induces metastasis, limiting its utility to studies of primary tumor growth rather than late-stage disease [151]. To recapitulate the native tumor microenvironment, luciferase-labeled cells (AGS-luc, 5 × 10⁶ cells/mouse in 25% Matrigel) are injected directly into the gastric submucosa via abdominal incision. Bioluminescent imaging enables non-invasive tracking of tumor growth, and this model reliably induces local invasion by 6–8 weeks, addressing the limitation of subcutaneous models. However, it requires surgical expertise, and tumor detection demands specialized imaging equipment [184]. For metastasis models, tail vein injection of 1 × 10⁶ cells studies hematogenous metastasis (predominantly to lungs and liver) [151, 185], while intraperitoneal injection of 3 × 10⁶−1 × 10⁷ cells induces peritoneal dissemination, mirroring the clinical pattern of advanced gastric cancer. Mice are euthanized when ascites or behavioral abnormalities emerge, typically 4–6 weeks post-injection, and metastatic nodules are quantified in abdominal organs (omentum, mesentery, diaphragm) [186, 187] (Fig. 7a).

Notably, CDX models have critical limitations. Long-term in vitro culture of cell lines erodes their genetic and phenotypic heterogeneity. Additionally, the absence of a functional human immune system (due to immunodeficient recipients) precludes studies of immunotherapies or tumor-immune microenvironment interactions. These constraints position CDX models as ideal for hypothesis-driven studies of cell-autonomous pathways or initial drug screening, rather than for predicting clinical responses to complex immunotherapies or microenvironment-targeted agents.

### Patient Derived Xenograft Mouse Model

PDX models occupy an irreplaceable niche in gastric cancer translational research by engrafting fresh human gastric tumor tissues into immunodeficient mice. This design preserves the native tumor microenvironment including stromal cells, vascular components, and extracellular matrix and retains the genetic, histological, and clinical heterogeneity of patient tumors, addressing a critical limitation of CDXs, which lose key biological features during long-term in vitro culture [188].

The establishment of PDX models follows a standardized yet clinically tailored workflow, with each step optimized to maintain tumor fidelity. Fresh tumor tissues are obtained from surgical resections or endoscopic biopsies (following ethical approval and patient consent), then immediately processed to remove non-tumor components (fat, connective tissue, normal gastric mucosa) using phosphate-buffered saline (PBS) supplemented with antibiotics to prevent microbial contamination [189]. Tissues are handled under sterile conditions, kept on ice during transport to minimize cell death, and cut into small fragments (≤ 150 μm in diameter) to facilitate nutrient exchange [190, 191]. These fragments are often mixed with Matrigel to enhance initial adherence and survival in the murine host. Immunodeficient mice typically NOD/SCID or NSG strains, selected for their low innate immunity and high human cell engraftment efficiency serve as recipients [190–192]. Implantation is either subcutaneous (for ease of monitoring tumor growth) or orthotopic (directly into the gastric submucosa via abdominal incision, to mimic the native anatomical microenvironment and better recapitulate invasion patterns) [193]. Once tumors reach a palpable size (usually 50–100 mm^3^) in the primary host (P1), they are excised, fragmented, and re-implanted into secondary mice (P2, P3, etc.) to expand the model and maintain consistent biological characteristics [189]. Fidelity validation is a critical step to confirm the model’s clinical relevance, as even minor genetic or phenotypic changes during passaging can undermine translational value. Post-engraftment, tumors are characterized using a combination of hematoxylin-eosin (H&E) staining, immunohistochemistry (IHC), and targeted gene sequencing or multi-omics analysis [191, 192, 194]. Only models that retain ≥ 80% similarity to the primary tumor across these metrics are deemed suitable for subsequent research, ensuring that experimental results are clinically interpretable (Fig. 7b).

PDX models are widely used to address unmet needs in gastric cancer research, with applications spanning drug development, personalized medicine, and mechanistic studies [195]. After PDX is established and validated, it can be passed for immune therapy, drug therapy experiments, virus targeting cancer gene treatment experiments, or metabolite treatment experiments. PDX models were established by subcutaneously implanting gastric cancer tissue fragments (10–50 mm³) or ascites cells (5 × 10⁵) into immunodeficient BALB/c NU/NU, NSG, or NOD/SCID mice [196]. When tumor volumes reached approximately 50–100 mm^3^, implanted mice were randomized into groups: a control group and experimental cohorts. Post-treatment analysis included flow cytometry and immunohistochemistry (IHC) to evaluate the treatment efficacy, while hematoxylin-eosin (H&E) staining of major organs (heart, liver, spleen, lung, kidney) assessed systemic toxicity [197–200]. The body weight stability throughout the study further corroborated treatment safety. These findings collectively validate the therapeutic efficacy and favorable safety profile of different therapies in this preclinical model. For example, irradiated CAR NK-92 cells (5 × 10⁶ cells per dose) were administered intravenously via the tail vein weekly for three consecutive weeks to study the efficacy of immune treatment [198]. The synergistic anti-tumor effect of dehydrocostus lactone (Dehy) and 5-FU was validated by intraperitoneal injection of Dehy (30 mg/kg) daily and 5-FU (25 mg/kg) every other day [199]. Intratumorally injection of si-CCAT5 (200 µg/kg) at a dose of 50 µL/time (to avoid fixed volume under physical pressure) was administered every 3 days to validate the anti-tumor effect of targeting CCAT5 ^172^. In addition, when the subcutaneous transplant tumor volume reached about 100 mm³, shSLC7A9 lentivirus was directly injected into the tumor tissue through multi-point injection, while the control group was injected with control virus (shNC), confirming that SLC7A9 knockdown significantly inhibited tumor cell proliferation [201].

PDX models have limitations. First, immunodeficient hosts lack a functional human immune system, precluding investigation of tumor-immune interactions unless additional humanization steps are performed and even humanized PDXs do not fully replicate the complexity of the human immune microenvironment. Second, biopsies yield limited tissue, and delayed processing (> 2 h post-resection) reduces engraftment rates. Third, genetic drift can occur during prolonged passaging, with loss of minor subclones that may drive drug resistance or metastasis. Finally, PDX models are resource intensive. They require longer tumor formation timelines (4–12 weeks) and higher costs (due to animal housing and tissue processing), limiting their scalability for large-scale drug screens. Despite these limitations, PDX models remain a gold standard for bridging preclinical research and clinical practice in gastric cancer.

## Conclusion

Gastric cancer remains a formidable global health challenge, necessitating robust preclinical models to elucidate pathogenesis and therapeutic strategies. New global data warn that *H. pylori* remains the dominant driver of gastric cancer, with a coming surge concentrated among young people and historically low-risk regions [202]. Thus, talk of a “post-*H. pylori* era” is premature. Our study updates *H. pylori*–co-factor models and, for the first time, systematically frames gastric cancers caused by non-*Helicobacter* microbes, closing yesterday’s gaps, defining today’s standards, and guiding tomorrow’s research. The review summarizes diverse methodologies for constructing gastric cancer mouse models, including chemical carcinogen-induced, microbe infection-induced, xenograft, and genetically engineered models, each tailored to address specific research questions. Chemical carcinogens such as MNU and MNNG effectively induce gastric tumors, offering reproducibility and scalability, albeit with prolonged latency. Their effectiveness can be limited by variability in dosage and strain sensitivity. *H. pylori*-driven models recapitulate human infection-associated carcinogenesis, with synergistic acceleration observed upon combining *H. pylori* with high-salt diets, alcohol, or chemical co-carcinogens. These models underscore the multifactorial nature of gastric cancer, aligning with epidemiological risk factors. *Streptococcus anginosus* infection has emerged as a novel factor in gastric cancer modeling, offering new avenues for research [14].

Genetically engineered mouse models (GEMMs) have revolutionized mechanistic studies by enabling precise manipulation of oncogenes (*Kras*, *Wnt*) and tumor suppressors (*Cdh1*, *Trp53*). Simultaneous knockout of tumor suppressor genes and activation of oncogenic drivers recapitulates the multi-stage progression of human gastric carcinogenesis. For spatiotemporal control of gene manipulation, inducible systems like Tet-On/Off or optogenetically regulated Cre-loxP permit tissue-specific like gastric antrum vs. corpus or temporally restricted like adult-stage mutagenesis, circumventing embryonic lethality while mimicking age-related cancer onset. Innovations like CRISPR/Cas9 and tissue-specific Cre-lox systems facilitate the exploration of gene-environment interactions and sequential carcinogenic events. Notably, models such as *INS-GAS* and *gp130*^F/F^ mice mimic chronic inflammation-driven tumorigenesis, while *Tff1* and *Muc6* knockouts highlight the role of mucosal integrity in cancer suppression.

Xenograft models, including cell-derived and patient-derived xenografts, are essential for preclinical testing of anticancer drugs. They preserve the tumor microenvironment and allow for the evaluation of drug efficacy in a setting that closely mimics human tumors. Particularly, patient-derived xenografts (PDXs) bridge translational gaps by preserving tumor heterogeneity and microenvironmental cues. To advance translational research in gastric cancer, humanized PDX models can be established by engrafting patient-derived peripheral blood mononuclear cells (PBMCs) or hematopoietic stem cells into immunodeficient mice [203]. These models enable precise evaluation of immunotherapy efficacy, such as immune checkpoint inhibitors, thereby expanding their utility in preclinical studies. Patient-derived organoids (PDOs) offer a high-throughput platform for rapid in vitro drug sensitivity screening [204]. By identifying therapeutic responders via PDO-based assays, researchers can prioritize sensitive cases for subsequent PDX model development, minimizing resource expenditure on non-responsive tumors [205]. Besides, to ensure genomic fidelity during PDX propagation, single-cell transcriptomic analysis should be employed to monitor tumor-stroma interactions and clonal evolution [206, 207]. This approach allows for the exclusion of models exhibiting genetic drift, preserving molecular characteristics that mirror the primary tumor. Subcutaneous and orthotopic CDX models offer simplicity and metastasis studies, respectively, whereas PDXs enable personalized therapeutic testing [208]. Emerging techniques, including bioluminescent tracking and CAR-T/NK cell therapies, enhance preclinical evaluations in these systems [209, 210].

In addition, we summarize core research rationales and objectives of various gastric cancer mouse models, encompassing chemical carcinogen-induced, *H. pylori* infection, *H. pylori*-combined factor, polymicrobial infection, genetically engineered, CDX, and PDX models. These models are developed to simulate diverse etiological scenarios of human gastric cancer, including environmental chemical exposure, primary *H. pylori* infection, synergistic interactions between infection and environmental/dietary/psychological factors, polymicrobial dysbiosis, genetic mutations, and preservation of human tumor cell/tissue characteristics, aiming to overcome limitations of single-factor models, enhance carcinogenesis efficiency, and reflect the complexity of real-world pathogenesis. Research objectives focus on elucidating fundamental carcinogenic mechanisms such as chemical/genetic pathways, inflammation-carcinogenesis crosstalk, and microbial-host metabolic interactions, developing diagnostic markers and preventive tools like vaccines and microbiota modulators, screening anticancer drugs, and validating personalized therapeutic strategies such as subtype-specific interventions, combined eradication/anti-inflammatory regimens, thereby bridging preclinical research with clinical translation for gastric cancer prevention, diagnosis, and treatment (Table 3). We have also systematically organized the core advantages, application constraints, and key experimental parameters of 7 core mouse strains in gastric cancer research, laying a foundation for the standardized construction of gastric cancer models and the cross-laboratory reproducibility of research results (Table 4).

**Table 3:** Research rationale and objectives of gastric cancer mouse models

Model Type	Reasons for Research	Research Objectives
Chemical carcinogen-induced model	1. Simulates environmental chemical exposure etiology, reproducing gastric cancer progression caused by dietary/pollutant factors; 2. Controllable experimental conditions with adjustable carcinogen dosage and exposure duration, ensuring standardized results; 3. Ethically and cost-effective with a short cycle (3–6 months), suitable for large-scale studies; 4. Mice share homologous digestive system with humans, and tumor pathological features are close to clinical cases.	1. Elucidate chemical carcinogenesis pathways and inflammation carcinogenesis associations; 2. Screen chemotherapeutic/targeted drugs and evaluate efficacy/resistance mechanisms; 3. Develop early diagnostic markers; 4. Validate chemopreventive strategies
*Helicobacter pylori* infection model	1. Simulates the primary biological etiology of gastric cancer, reproducing the natural infection-chronic inflammation-carcinogenesis process (Correa cascade); 2. Regulable bacterial virulence to study interactions between bacterial virulence factors and host; 3. Standardized experimental conditions; 4. Lower cost than primates, suitable for long-term infection studies (6–12 months).	1. Decipher carcinogenic mechanisms of CagA/VacA and IL-6/STAT3 inflammatory signaling; 2. Screen antibiotic eradication regimens and virulence factor-targeted drugs; 3. Develop vaccines and diagnostic markers; 4. Validate combined eradication and anti-inflammation preventive strategies.
*H. pylori* combined factor models (*H. pylori* + chemicals/alcohol/high salt/capsaicin/stress)	1. Simulates human infection combined with environmental/dietary/psychological factors synergistic carcinogenesis; 2. Improves carcinogenesis efficiency and shortens the cycle; 3. Reveals multi-factor interaction mechanisms.	1. Elucidate synergistic carcinogenic pathways of infection combined with chemical damage/diet/stress; 2. Develop stratified prevention strategies for high-risk populations; 3. Screen multi-target drugs; 4. Simulate clinical multi-etiology backgrounds to optimize personalized treatment.
Polymicrobial infection model	1. Simulates gastric dysbiosis-induced carcinogenesis; 2. Studies microbiota-driven carcinogenesis via metabolites and virulence factor interactions; 3. Compensates for the limitation of single *H. pylori* infection in simulating comprehensive microbiota metabolic effects.	1. Decipher synergistic pathways between *H. pylori* and pro-carcinogenic bacteria; 2. Identify pro-carcinogenic microbiota markers; 3. Validate probiotic (*Lactobacillus*) microbiota modulation for cancer prevention.
Genetically engineered model	1. Simulates genetic driver carcinogenesis by directly recapitulating human gastric cancer mutations via gene editing; 2. Controllable genetic background to exclude interfering factors; 3. Models specific subtypes.	1. Elucidate carcinogenic pathways of tumor suppressor inactivation, oncogene activation, and inflammatory gene overexpression; 2. Screen mutation-targeted drugs and resistance mechanisms; 3. Validate gene therapy and cell therapy feasibility; 4. Develop gene-signature-based early diagnostic markers.
Cell-derived xenograft (CDX) model	1. Preserves biological characteristics of human gastric cancer cell lines; 2. Stable genetic background supporting gene editing for high reproducibility; 3. Short tumor formation cycle (2–4 weeks), suitable for large-scale drug screening with lower cost than PDX models.	1. Study tumor cell proliferation, metastasis, and drug resistance mechanisms; 2. In vivo efficacy screening of anticancer drugs; 3. Evaluate targeting and safety of nanodrug delivery systems.
Patient-derived xenograft (PDX) model	1. Preserves patient tumor heterogeneity and clinical behavior; 2. Drug sensitivity consistent with patient clinical responses, enabling personalized drug sensitivity testing; 3. Stable long-term passaging to support subtype-genotype-phenotype PDX biobank construction.	1. Decipher tumor heterogeneity evolution, microenvironmental crosstalk, and metastasis “seed-soil” mechanisms; 2. Screen personalized treatment regimens; 3. Accelerate preclinical evaluation of novel drugs and validate combination therapies; 4. Identify efficacy-predictive markers.

**Table 4:** Characteristics of common mouse strains for gastric cancer research

Mouse Strain	Main Advantages	Constraints	Bacterial Colonization (Key Pathogens)	Survival Rate (Typical Modeling Period)	Tumorigenesis Timeline (Key Models)
C57BL/6J	1. Well-defined genetic background, abundant transgenic/knockout derivatives; 2. Moderate sensitivity to *H. pylori* and chemical carcinogens; 3. High reproducibility, widely used in inflammation-carcinogenesis studies.	1. Low tumor incidence with single *H. pylori* infection; 2. Requires co-factors (e.g., MNU, high salt) to accelerate tumorigenesis.	*H. pylori* (PMSS1 strain): 85–95% colonization rate; *H. felis*: 90–100% colonization rate; *S. anginosus*: 70–80% (needs continuous gavage).	> 90% (12–18 months for infection models; 6–12 months for chemical-co models)	Single *H. pylori*: 40–50% adenocarcinoma at 18th month; *H. pylori* & MNU: >70% tumors at 12th month; MNU alone: 60–70% tumors at 54th week.
BALB/c	1. Stable response to *H. pylori* infection, suitable for chronic gastritis models; 2. High survival rate under combined chemical-infection protocols.	1. Low sensitivity to MNNG (requires higher doses for lesion induction); 2. Difficulty in forming tumors with single *H. pylori* infection.	*H. pylori* (SS1 strain): 70–80% colonization rate; *H. suis*: 65–75% colonization rate.	> 85% (10–16 months for combined models)	*H. pylori* & MNU: Precancerous lesions (CAG/IM) at 20th week; MNNG alone (250 µg/mL): CAG at 12th week, tumors at 38th week.
Mongolian Gerbil	1. Highest sensitivity to *H. pylori* among rodent models; 2. Spontaneous gastric cancer formation with single *H. pylori* infection (unique to this strain).	1. Long modeling timeline; 2. Higher cost than mice; 3. Limited genetic manipulation tools.	*H. pylori* (ATCC43504 strain): ~100% colonization rate; *H. pylori* (TN2GF4 strain): 95–100% colonization rate.	70–80% (> 62 weeks for single infection)	Single *H. pylori*: ~37% gastric cancer at 62nd week; *H. pylori* & MNU: 60–75% tumors at 52nd week; *H. pylori* & high salt: 63% tumors at 50th week (10% NaCl diet).
FVB	1. High fecundity, suitable for transgenic model generation (e.g., INS-GAS); 2. Sensitive to *H. felis*-induced hypergastrinemia.	1. Low sensitivity to chemical carcinogens (e.g., MNU); 2. Poor *H. pylori* colonization (SS1 strain: <60%).	*H. felis*: 85–95% colonization rate; *H. pylori* (SS1 strain): <60% colonization rate.	> 90% (6–12 months for transgenic models)	INS-GAS & *H. felis*: 85% mucosal carcinoma at 6–7th month; INS-GAS alone: Gastric atrophy at 5th month, tumors at 20th month.
CF1	1. High sensitivity to combined chemical carcinogens (DEN & NNN & ethanol); 2. Rapid tumor formation, suitable for short-term efficacy studies.	1. Limited genetic background information; 2. Not applicable for genetic manipulation (e.g., crossbreeding with transgenic strains).	*H. pylori*: Not commonly used (colonization rate < 50%); No reported data for other pathogens.	80–85% (24 weeks for chemical models)	DEN & NNN & ethanol: ~85% gastric cancer at 24th week.
NSG (NOD-SCID-IL2Rγ⁻/⁻)	1. Severe immunodeficiency, highest engraftment rate for human tumor cells/tissues; 2. Suitable for PDX/CDX models, supports long-term tumor growth.	1. Lack of functional immune system, cannot study infection-immune crosstalk; 2. High cost, requires strict SPF conditions.	Not used for *H. pylori*/microbe models (no immune response); Human gastric cancer cells: ~100% engraftment.	> 90% (2–6 months for xenograft models)	Subcutaneous CDX (MKN-45): Tumor formation at 2–3rd week; Orthotopic PDX: Tumor formation at 4–8th week, metastasis at 12–16th week.
NOD/SCID	1. Moderate immunodeficiency, suitable for CDX models; 2. Lower cost than NSG, widely used in preliminary drug screening.	1. Residual natural killer cell activity, lower engraftment rate than NSG; 2. Not ideal for long-term PDX passaging.	Human gastric cancer cells (e.g., AGS): 85–90% engraftment; Microbe models: Not applicable.	85–90% (3–5 months for xenograft models)	Subcutaneous CDX (AGS): Tumor formation at 3–4th week; Intraperitoneal CDX (YTN16): Peritoneal dissemination at 4–6th week.
BALB/c nu/nu (Nude Mouse)	1. T-cell deficiency, low cost, easy to maintain; 2. Suitable for basic CDX models (e.g., primary tumor growth studies).	1. B-cell and NK cell activity remain, low engraftment rate for metastatic cell lines; 2. Not suitable for PDX or infection models.	Human gastric cancer cells (e.g., SGC-7901): 75–85% engraftment; Microbe models: Not applicable.	80–85% (3–6 months for CDX models)	Subcutaneous CDX: Tumor formation at 4–5th week; No reported spontaneous metastasis.

Bacterial Colonization: Focuses on *H. pylori* (the primary carcinogen) and other key pathogens (e.g., *H. felis, S. anginosus*) mentioned in the original manuscript; colonization rates are derived from standardized protocols (oral gavage of 1×10⁸−1×10⁹ CFU/mL, 3–5 doses)

Survival Rate: Refers to the percentage of mice surviving the full modeling period (excluding euthanasia for time-point analysis) under standard husbandry conditions

Tumorigenesis Timeline: Prioritizes widely used models (e.g., *H. pylori alone, H. pylori* & co-factors, chemical carcinogens, xenografts) to align with the core content of the review

Despite progress, challenges persist. *H. pylori* models require optimization to reduce variability, while GEMMs demand improved clinical relevance. PDXs face scalability and stromal interaction limitations [211]. Future efforts should integrate multi-omics and organoid technologies to refine model fidelity and accelerate drug discovery. Collaborative innovation across disciplines will be pivotal in advancing these models, ultimately translating mechanistic insights into effective clinical interventions. In summary, the strategic selection and refinement of gastric cancer mouse models guided by experimental objectives and clinical parallels will remain indispensable in unraveling disease complexity and developing targeted therapies.

## Data Availability

No datasets were generated or analysed during the current study.

## Abbreviations

*H. pylori*: *Helicobacter pylori*
EBV: Epstein-Barr virus
MNU: N-methyl-N-nitrosourea
MNNG: N-methyl-N'-nitro-N-nitrosoguanidine
3-MCA: 3-methylcholanthrene
DEN: Diethylnitrosamine
NNN: N'-nitrosonornicotine
*H. felis*: *Helicobacter felis*
WIRS: Water-immersion restraint stress
H&E: Hematoxylin and eosin
*S. anginosus*: *Streptococcus anginosus*
GEMMs: Genetically engineered mouse models
CEA: Carcinoembryonic antigen
EGFR: Epidermal growth factor receptor
SPEM: Spasmodic peptide-expressing metaplasia
IM: Intestinal metaplasia
CAG: Chronic atrophic gastritis
HGIN: High-grade intraepithelial neoplasia
INS-GAS: Insulin-Gastrin
COX-2: Cyclooxygenase-2
mPGES-1: microsomal prostaglandin E synthase 1
LKB1: Liver kinase B1
PTEN: Phosphatase and tensin homolog
EMT: Epithelial-mesenchymal transition
PGE2: Prostaglandin E2
K19: Keratin 19
Kras: Kirsten rat sarcoma viral oncogene homolog
GA: Gastric adenocarcinoma
Tcon: Triple conditional knockout
Dcon-Ecad het: Dual conditional E-cadherin heterozygous
GC: Gastric cancer
CDX model: Cell derived xenograft mouse model
SCID: Severe Combined Immunodeficiency
IP: Intraperitoneally
IACUC: Institutional Animal Care and Use Committee
PDX model: Patient derived xenograft mouse model
IHC: Immunohistochemistry
PBMCs: Patient-derived peripheral blood mononuclear cells
PDOs: Patient-derived organoids
NSG mice: NOD-SCID gamma mice
